# Traditional Uses, Chemical Constituents, Biological Properties, Clinical Settings, and Toxicities of *Abelmoschus manihot* L.: A Comprehensive Review

**DOI:** 10.3389/fphar.2020.01068

**Published:** 2020-08-26

**Authors:** Fei Luan, Qianhong Wu, Yan Yang, Haizhen Lv, Daoheng Liu, Zhaoping Gan, Nan Zeng

**Affiliations:** ^1^ Department of Clinical Pharmacy, Shaanxi Provincial Hospital of Tuberculosis Prevention and Treatment, Xi’an, China; ^2^ Department of Pharmacology, College of Pharmacy, Chengdu University of Traditional Chinese Medicine, Chengdu, China; ^3^ Department of Bioengineering, Zhuhai Campus of Zunyi Medical University, Zhuhai, China

**Keywords:** *Abelmoschus manihot* L., traditional uses, total flavones, antidiabetic nephropathy activity, clinical settings, toxicological

## Abstract

*Abelmoschus manihot*, an annual herbal flowering plant, is widely distributed throughout eastern Europe and in temperate and subtropical regions of Asia. Its flowers have been traditionally used for the treatment of chronic kidney disease in China. Currently, more than 128 phytochemical ingredients have been obtained and identified from the flowers, seeds, stems, and leaves of *A. manihot*. The primary components are flavonoids, amino acids, nucleosides, polysaccharides, organic acids, steroids, and volatile oils. *A. manihot* and its bioactive constituents possess a plethora of biological properties, including antidiabetic nephropathy, antioxidant, antiadipogenic, anti-inflammatory, analgesic, anticonvulsant, antidepressant, antiviral, antitumor, cardioprotective, antiplatelet, neuroprotective, immunomodulatory, and hepatoprotective activities, and have effects on cerebral infarction, bone loss, etc. However, insufficient utilization and excessive waste have already led to a rapid reduction of resources, meaning that a study on the sustainable use of *A. manihot* is urgent and necessary. Moreover, the major biologically active constituents and the mechanisms of action of the flowers have yet to be elucidated. The present paper provides an early and comprehensive review of the traditional uses, chemical constituents, pharmacological activities, and pharmaceutical, quality control, toxicological, and clinical settings to emphasize the benefits of this plant and lays a solid foundation for further development of *A. manihot*.

## Introduction

**Figure 1:**
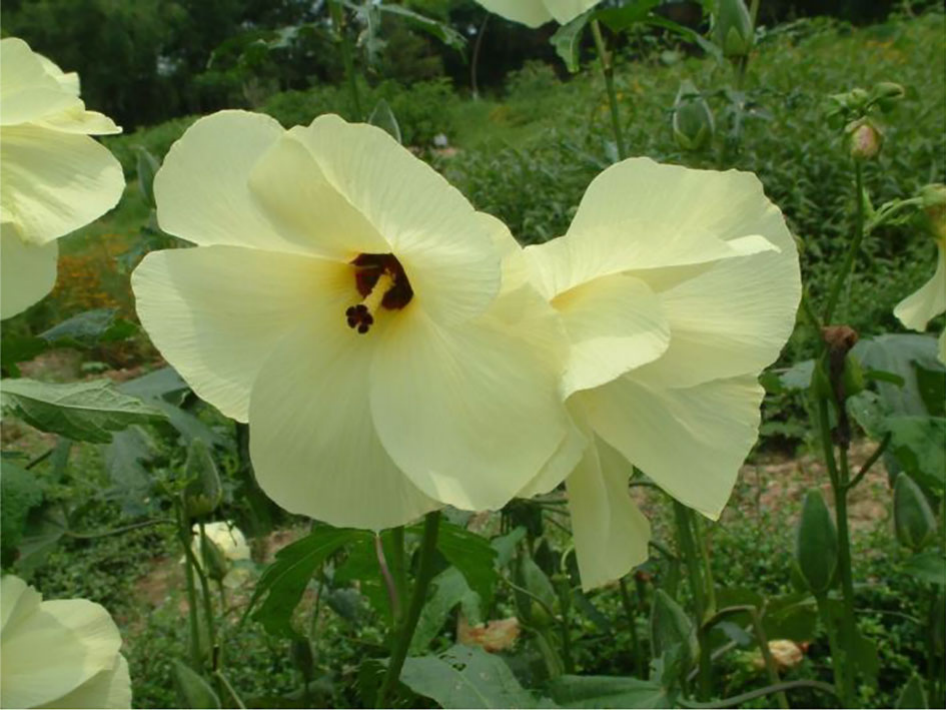
Photograph of *Abelmoschus manihot* (L.) Medicus. Courtesy of Huachun Xu, Lengshui, Bijie, Guizhou Province of China.

*Abelmoschus manihot* (L.) Medicus (syn.: *Hibiscus manihot*; **Figure 1**), which belongs to the Malvaceae family and is commonly called *Huang Shu Kui Hua* (in Chinese), *Dakpul* (in Korean), and *Aibika* (in Indonesian), is an annual or perennial herbaceous flowering plant and an edible form of hibiscus. It is distributed widely throughout eastern Europe and Asia, including China, Papua New Guinea, eastern Indonesia, Nepal, Fiji, India, Sri Lanka, Vanuatu, New Caledonia, and northern Australia (Park et al., 2015; Prabawardani et al., 2016; Rubiang-Yalambing et al., 2016). *A. manihot*, which has been demonstrated to have high nutritional value for human health, is commonly used as a green vegetable and is very popular in the South Pacific Islands, Papua New Guinea, and eastern Indonesia (Prabawardani et al., 2016; Rubiang-Yalambing et al., 2016). In China, numerous health foods have been commercially developed using the roots, stems, and leaves of *A. manihot* (Du L. Y. et al., 2015; Du L. et al., 2015). For hundreds of years, *A. manihot* has been widely used as a folk medicine in clinical practice in China to cure forms of chronic kidney disease (CKD) and exerts significant effects by decreasing the protein content in urine and protecting kidney function (Peng et al., 2010; Wang and Gao, 2010; Zhu and Bi, 2010; Tsumbu et al., 2012). According to the *Compendium of Materia Medica*, a famous classical book of Chinese materia medica compiled by Shizhen Li (1518–1593 CE), the flowers of *A. manihot* have been recorded as an effective herb to treat malignant sores, cellulitis, and burns (Jiang et al., 2012; Cai et al., 2017a; Hou et al., 2020). Importantly, *A. manihot* flowers are listed in the 2015 edition of the *Pharmacopoeia of the People’s Republic of China* (a.k.a. the *Chinese Pharmacopoeia*) for treating many diseases, such as chronic glomerulonephritis and diabetic nephropathy (DN), in clinical practice. The *Chinese Pharmacopoeia* states that the content of hyperoside, which is used as a standard for quality control of *A. manihot* and its compound preparations, in *A. manihot* flowers should not be less than 0.5% (Guo et al., 2013; Committee for the Pharmacopoeia of P.R. China, 2015). Furthermore, Huangkui capsule (HKC), a Chinese patent medicine, is a single plant-based drug extracted from the dry flowers of *A. manihot*. HKC was approved by the State Food and Drug Administration of China (Z19990040) in 1999 for the treatment of CKD, such as DN, chronic glomerulonephritis, membranous nephropathy, and other inflammatory diseases, in clinical practice (Zhang et al., 2014; Du L. Y. et al., 2015; Li et al., 2017). Its main mechanisms of action include improving the immune response, decreasing inflammation, improving renal fibrosis, and protecting renal tubular epithelial cells (Chen et al., 2012).

Modern pharmacological findings have shown that the extracts and active constituents of *A. manihot* possess various biological properties, including anti-DN (Tu et al., 2013), anticonvulsant (Guo et al., 2011), antioxidant (Zhang et al., 2013), antiadipogenic, anti-inflammatory (Jain and Bari, 2010), analgesic (Fan et al., 2003), antidepressant (Guo et al., 2011), antiviral (Wu et al., 2007), antitumor (Zheng et al., 2016), antiplatelet (Guo et al., 2005), anti-Crohn’s disease (Yang B. L. et al., 2018), anti-poststroke depression (Liu et al., 2009), proangiogenic (Zhu et al., 2018), cardioprotective (Lv et al., 2017), neuroprotective (Cheng et al., 2006), immunomodulatory (Pan et al., 2018), and hepatoprotective (Ai et al., 2013) properties, and are effective against cerebral infarction, bone loss (Guo and Chen, 2002; Puel et al., 2005), etc. Many of these activities are consistent with those of *A. manihot* in traditional medicine and support traditional usage. Overall, these investigations summarize the pharmacological activities of *A. manihot* flowers in many medical situations.

Because of their marked clinical therapeutic effects and nutritional value, increasing numbers of researchers have widely and intensively studied the chemical components of the flowers of *A. manihot*. Phytochemical investigations found that flavonoids, amino acids, nucleosides, polysaccharides, organic acids, steroids, and volatile oils were the main components present in the flowers, seeds, stems, and leaves of *A. manihot*. Among these, flavonoids have been regarded as the active components and are officially used as markers to monitor the quality of the herb and preparations containing extracts of *A. manihot* according to the 2015 edition of the *Chinese Pharmacopoeia*; therefore, flavonoids have been the most widely studied components (Chi et al., 2009; Yu et al., 2014; Xia et al., 2019). However, conclusions about the pharmacological activities of a majority of bioactive compounds derived from traditional Chinese Medicine or natural products have been strongly questioned in the literature on pan-assay interference compounds (PAINS) (Baell and Walters, 2014; Baell and Nissink, 2018; Heinrich et al., 2020). As a result, these flavonoids should be assessed and distinguished in the future.

In recent decades, many attempts have been made to investigate the phytochemistry and pharmacology of *A. manihot*. Several authors have published reviews regarding the chemical constituents and pharmacological effects of *A. manihot* components only (Liu et al., 2010; Todarwal et al., 2011; Wen et al., 2015). However, the published works do not contain comprehensive and up-to-date information on *A. manihot*. We have therefore reviewed and discussed in detail the complete range of recent advances in scientific information on the botanical details, traditional uses, chemical constituents, pharmacological activities, clinical settings, pharmacokinetics and metabolism, qualitative and quantitative analysis, and toxicity of *A. manihot* to support its therapeutic potential. We believe this article will be a guide for the full utilization of this plant in the development of novel candidate drugs and therapies for various diseases, especially CKD.

## Botanical Characterization

*A. manihot* is widely distributed in valleys and grasslands, at the edges of fields, and in or near ditches. It is 1–2 m high and the whole plant is sparsely hirsute. The leaves are nearly circular, with five to nine palmate lobes, and are 10–30 cm in diameter; the lobes are oblong–lanceolate, 8–18 cm long, and 1–6 cm wide with an acuminate apex and coarse blunt teeth; the length of the petiole is 6–20 cm; and the stipules are lanceolate and 0.8–1.5 cm long. The flowers are solitary and borne on the axils of terminal leaves on the branches. The pedicel is 1–3 cm long; there are four or five ovate–lanceolate bracts that are 1.2–2.5 cm in length, 0.4–1 cm wide, and sparsely hirsute; the calyx is spathulate, subentire, and apically five-toothed; and the smaller bracts are slightly longer and pubescent and are shed with the fruit. The corolla is funnel-shaped and yellow with a purple inner surface at the base and is 7–12 cm in diameter; there are five petals, which are broadly obovate; the stamen columns are 1.2–2 cm in length with basal anthers, which are subsessile; the ovary has five chambers, each of which contains multiple ovules; the style has five branches; and the stigma is purple and in the form of a spoon-shaped disk. The capsule is ovate–elliptical, covered in bristles, 4–6 cm long, and 2–3 cm in diameter, and the length of the stalk is 8 cm. Most seeds are kidney-shaped with several vertical stripes of pubescence. The plant flowers from July to October (http://ppbc.iplant.cn/sp/22499; Chen et al., 2016).

## Traditional Uses

Traditional Chinese medicine has served the Chinese people for 2000 years, preventing and treating diseases and maintaining health; it remains an important part of the provision of medical services (Dhiman and Chawla, 2005; Stickel and Schuppan, 2007; Yan et al., 2015). Currently, attracted by herbal medicines’ properties of high efficiency, low toxicity, and low cost with respect to chemical drugs, increasing numbers of scientists are turning to supplementary medicine with great interest to study kidney-protecting agents (Yan et al., 2015).

In ancient China, *A. manihot* was first described in the two oldest books on classical medicine; namely, *Jia You Ben Cao* (simplified Chinese: 嘉佑本草) and *Ban Cao Gang Mu* (simplified Chinese: 本草纲目). It was officially listed in the 2015 edition of the *Chinese Pharmacopoeia* because of its medical and economic value and because it is widely distributed in many provinces of China, including Shanxi, Guangdong, Fujian, Guizhou, Guangxi, Hebei, Henan, Hubei, Hunan, Shandong, Sichuan, Yunnan, and Taiwan (Yan et al., 2015; Chen et al., 2016). It is described as sweet and cold in nature, and it acts on the kidney and bladder meridians (Committee for the Pharmacopoeia of P.R. China, 2015). The flowers, roots, leaves, and seeds of *A. manihot* have traditionally been widely used to treat edema and damp heat and to heal ulcers by expelling toxins; they have also been used to treat burns due to water and fire (Committee for the Pharmacopoeia of P.R. China, 2015; Pan et al., 2018). In particular, since the Song dynasty (960–1279 BC) the flowers of *A. manihot* have been widely regarded as a staple food and folk medicine in China, and have been used to alleviate inflammation, protect against kidney injuries, and restore tissue affected by ulcers and burns (Tu et al., 2013; Zhang et al., 2014). In addition, the flowers of *A. manihot*, when administered in the form of a healthy beverage, have been proved to possess a wide range of pharmacological properties such as activity against DN and CKD (Zhou et al., 2012; Du et al., 2017; Wan et al., 2019). In addition, a decoction of *A. manihot* flowers is traditionally used to treat jaundice and acute and chronic hepatitis in the Anhui and Jiangsu Provinces of China (Wang et al., 2004). Importantly, HKC has been widely used for many years in clinical practice to effectively treat chronic glomerulonephritis (Tu et al., 2013; Mao et al., 2015; Ge et al., 2016), alleviate proteinuria, and relieve kidney insufficiency in patients with early-stage CKD in clinical practice (Han and Qiu, 2010; Liu et al., 2011). Simultaneously, many functional foods have been obtained from the roots, stems, and leaves of *A. manihot* because of their abundant nucleotides, nucleosides, and nucleobases (Du L. Y. et al., 2015). Furthermore, nucleotides, nucleosides, and nucleobases have been regarded as quality control markers for many traditional Chinese medicines, such as royal jelly, *Mactra veneriformis*, and *Cordyceps sinensis* (Liu R. et al., 2012; Wu et al., 2015; Lin et al., 2018; Zhou et al., 2018).

*A. manihot* is mainly distributed in tropical areas, especially Asia and the Pacific Islands. Its flowers and leaves are edible and have medicinal effects, and they have been traditionally used to treat inflammation, pain, urinary infections, and chronic bronchitis because of their anti-inflammatory, antiviral, antibacterial, and wound-healing properties (Yang et al., 2015; Rubiang-Yalambing et al., 2016; Kim et al., 2018). In eastern Indonesia, the South Pacific Islands, and Papua New Guinea, *A. manihot* is regarded as an edible hibiscus and is consumed as a popular leafy vegetable because it contains high levels of nutrients such as Ca, Fe, K, Mg, Mn, Na, Zn, Cu, folate, and β-carotene (Rubiang-Yalambing et al., 2016). In particular, in Papua New Guinea and the Pacific Islands, the flower of *A. manihot* is employed as a staple folk medicine to alleviate kidney pain, lower high cholesterol levels, impede menorrhagia, induce abortions, ease childbirth, stimulate lactation, treat diarrhea, and prevent osteoporosis (Bourdy and Walter, 1992; Puel et al., 2005; Prabawardani et al., 2016). Interestingly, as a green leafy vegetable *A. manihot* is an integral part of the main daily meal in rural and urban areas of Papua New Guinea, and approximately 75% of local people consume this vegetable because it is rich in micronutrients (Tsumbu et al., 2011). In India, *A. manihot* is used as a source of traditional medicines for treating kidney pain, osteoporosis, high cholesterol levels, and heartburn (Todarwal et al., 2011). In Nepal, the juice from the leaves and roots of *A. manihot*, which has remarkable analgesic effects, is commonly and traditionally used for the treatment of sprains (Todarwal et al., 2011; Taroreh et al., 2016). In Korea, *A. manihot* has been used to make the traditional form of paper known as hanji. It was previously recognized as a species of hibiscus, although it is now classified in the genus *Abelmoschus* and its scientific name is *Abelmoschus manihot* (Kim et al., 2018).

In African states such as the Democratic Republic of the Congo, Cameroon, Uganda, Nigeria, Gabon, and Angola, *A. manihot* is very important in the local economy because it is the principal green vegetable for local residents in these nations (Almazan and Theberge, 1989; Kubo et al., 2006). The seeds and leaves of *A. manihot* have traditionally been used in Africa to treat rheumatism, fever, headache, and hemorrhoids in folk medicine (Miladiyah et al., 2011). In particular, *A. manihot* is utilized for the treatment of ringworm, tumors, conjunctivitis, sores, and abscesses in Nigeria (Miladiyah et al., 2011).

## Chemical Constituents

**Figure 2:**
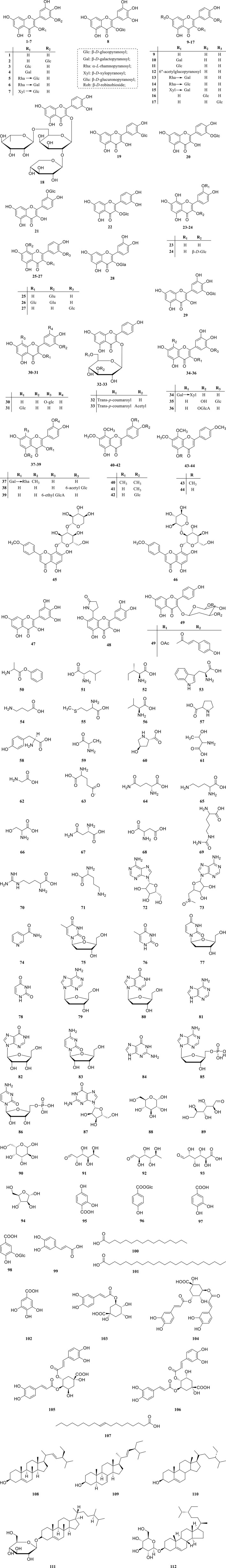
Structure formulae of chemical compounds isolated from *Abelmoschus manihot* L.

According to the available literature, approximately 128 chemical constituents have been isolated from *A. manihot*, most of which were purified from the flowers. Here, these constituents are classified into eight groups; namely, flavonoids, amino acids, nucleosides, polysaccharides, organic acids, steroids, volatile oils, and others (**Table 1** and **Figure 2**). We also list five reported polysaccharides obtained from *A. manihot* and provide comprehensive information with regard to their molecular weights, monosaccharide compositions, structural features, and bioactivities, as well as associated references, in **Table 2**.

**Table 1:** The chemical constituents obtained and identified from *Abelmoschus manihot*.

No.	Chemical constituent	Extract	Part	Reference
**Flavonoids**				
1	Myricetin	EtOH	Flowers	Lai et al., 2006
		EtOH	Flowers	Zhang et al., 2008
		EtOH	Flowers	Li et al., 2010a
		EtOH	Flowers	Li et al., 2016
		EtOH	Flowers	Pan et al., 2017
2	Myricetin-3-*O*-β-D-glucopyranoside	EtOH	Flowers	Lai et al., 2006
		EtOH	Flowers	Li et al., 2010a
3	Myricetin-3-*O*-glucoside	EtOH	Flowers	Zhang et al., 2008
4	Myricetin-3-*O*-β-D-galactopyranoside	EtOH	Flowers	Li et al., 2010a
		EtOH	Flowers	An et al., 2011
5	Myricetin-3-*O*-rutinose	EtOH	Flowers	Li et al., 2010a
6	Myricetin-3-*O*-robinoside	EtOH	Flowers	Li et al., 2010a
		EtOH	Flowers	An et al., 2011
7	Myricetin-3-*O*-β-D-xylopyranosyl-(1→2)-β-D-glucopyranoside	EtOH	Flowers	Li et al., 2010a
		EtOH	Flowers	An et al., 2011
8	Myricetin-3-*O*-β-D-glucoside	EtOH	Flowers	Chen, 2006
9	Quercetin	EtOH	Flowers	Zhang et al., 2008
		EtOH	Flowers	Li et al., 2010b
10	Quercetin-3′-β-glucoside	EtOH	Flowers	Zhang et al., 2008
11	Quercetin-3-*O*-β-D-glucopyranoside	EtOH	Flowers	Li et al., 2010b
12	Quercetin-3-*O*-β-D-6″-acetylglucopyranoside	EtOH	Flowers	Li et al., 2010b
13	Quercetin-3-*O*-robinoside	EtOH	Flowers	Li et al., 2010b
		EtOH	Flowers	An et al., 2011
14	Quercetin-3-*O*-rutinoside	EtOH	Flowers	Li et al., 2010b
		EtOH	Flowers	Li et al., 2016
		EtOH	Flowers	Yang Z. Z. et al., 2018
15	Quercetin-3-*O*-β-D-xylopyranosyl-(1→2)-β-D-galactopyranoside	EtOH	Flowers	Li et al., 2010b
		EtOH	Flowers	An et al., 2011
16	Quercetin-3′-*O*-β-D-glucopyranoside	EtOH	Flowers	Li et al., 2010b
		EtOH	Flowers	Li et al., 2016
		EtOH	Flowers	Yang Z. Z. et al., 2018
17	Quercetin-7-*O*-β-D-glucopyranoside	EtOH	Flowers	Li et al., 2010b
		EtOH	Flowers	An et al., 2011
18	Quercetin-3-*O*-[β-D-xylopyranosyl(1→2)-α-L-rhamnopyranosyl-(1→6)-β-D-galactopyranoside	EtOH	Flowers	Li et al., 2011
19	Quercetin-3′-glucoside	EtOH	Flowers	Wang et al., 2004
		EtOH	Flowers	Wang et al., 1981
20	Quercetin-3-*O*-β-robinobioside	EtOH	Flowers	Wang et al., 1981
		EtOH	Flowers	Chen, 2006
21	Quercetin-3′-*O*-β-D-glucoside	EtOH	Flowers	Chen, 2006
22	Isoquercitrin	EtOAc	Flowers	Chen et al., 2007
		EtOH	Flowers	Li et al., 2016
		EtOH	Flowers	Yang Z. Z. et al., 2018
23	Gossypetin	EtOH	Flowers	Chen, 2006
24	Gossypetin-3′-*O*-β-D-glucoside	EtOH	Flowers	Chen, 2006
25	Gossypetin-8-*O*-β-D-glucuronide	EtOH	Flowers	Li et al., 2011
26	Gossypetin-3-*O*-β-glucopyranoside-8-O-β-glucuronopyranoside	EtOH	Flowers	Li et al., 2011
27	Gossypetin-3’-*O*-β-D-glucopyranoside	EtOH	Flowers	Li et al., 2011
		EtOH	Flowers	Wang et al., 2004
28	Hyperoside	EtOH	Flowers	Zhang et al., 2008
		EtOH	Flowers	Li et al., 2016
		EtOH	Flowers	Fan et al., 2011
		EtOH	Flowers	Li et al., 2010b
		EtOH	Flowers	Yang Z. Z. et al., 2018
29	Cannabiscitrin	EtOH	Flowers	Lai et al., 2006
30	Hibifolin	EtOH	Flowers	Li et al., 2016
31	Hibiscetin-3-*O*-glucoside	EtOH	Flowers	Zhang et al., 2008
32	Tiliroside	EtOH	Flowers	Li et al., 2011
33	Kaempferol-3-*O*-[3″-*O*-acetyl-6″-*O*-(E)-p-coumaroyl)]-β-D-glucopyranoside	EtOH	Flowers	Li et al., 2011
34	Floramanoside A	EtOH	Flowers	Zhang et al., 2013
35	Floramanoside B	EtOH	Flowers	Zhang et al., 2013
36	Floramanoside C	EtOH	Flowers	Zhang et al., 2013
37	Floramanoside D	EtOH	Flowers	Zhang et al., 2013
38	Floramanoside E	EtOH	Flowers	Zhang et al., 2013
39	Floramanoside F	EtOH	Flowers	Zhang et al., 2013
40	5-Hydroxyl-3′,4′,7,8-tetramethoxyl flavone	EtOH	Flowers	Chen, 2006
41	3′,5-Dihydroxyl-4′,7, 8-trimethoxyl flavone	EtOH	Flowers	Chen, 2006
42	3′,5-Dihydroxyl-7,8-dimethoxyl flavone-4′-*O*-β-D-glucoside	EtOH	Flowers	Chen, 2006
43	4′,5,7,8-Tetramethoxyl flavone	EtOH	Flowers	Chen, 2006
44	5-Hydroxyl-4′,7,8-trimethoxyl flavone	EtOH	Flowers	Chen, 2006
45	4′-Methoxyl-5,7-dihydroxyl flavone-[-*O*-β-D-xylopyranosyl-(1→3)]-*O*-β-D-glucopyranoside	EtOH	Flowers	Chen, 2006
46	4′,7-Dimethoxyl-5,7-dihydroxyl flavone-[-*O*-β-D-xylopyranosyl-(1→2)]-*O*-β-D-glucopyranoside	EtOH	Flowers	Chen, 2006
47	Dihydromyricetin	MeOH	Flowers	Yang et al., 2017
48	8-(2″-pyrrolidinone-5-yl)-quercetin	MeOH	Flowers	Yang et al., 2017
49	3-*O*-kaempferol-3-*O*-acetyl-6-*O*-(*p*-coumaroyl)-α-D-glucopyranoside	MeOH	Flowers	Yang et al., 2017
**Amino acids**				
50	Phenylalanine	Aqueous	Roots, stems, and leaves	Du L. et al., 2015
51	Leucine	Aqueous	Roots, stems, and leaves	Du L. et al., 2015
52	Isoleucine	Aqueous	Roots, stems, and leaves	Du L. et al., 2015
53	Tryptophan	Aqueous	Roots, stems, and leaves	Du L. et al., 2015
54	γ-Aminobutyric acid	Aqueous	Roots, stems, and leaves	Du L. et al., 2015
55	Methionine	Aqueous	Roots, stems, and leaves	Du L. et al., 2015
56	Valine	Aqueous	Roots, stems, and leaves	Du L. et al., 2015
57	Proline	Aqueous	Roots, stems, and leaves	Du L. et al., 2015
58	Tyrosine	Aqueous	Roots, stems, and leaves	Du L. et al., 2015
59	Alanine	Aqueous	Roots, stems, and leaves	Du L. et al., 2015
60	Hydroxyproline	Aqueous	Roots, stems, and leaves	Du L. et al., 2015
61	Threonine	Aqueous	Roots, stems, and leaves	Du L. et al., 2015
62	Glycine	Aqueous	Roots, stems, and leaves	Du L. et al., 2015
63	Glutamate	Aqueous	Roots, stems, and leaves	Du L. et al., 2015
64	Glutamine	Aqueous	Roots, stems, and leaves	Du L. et al., 2015
65	Lysine	Aqueous	Roots, stems, and leaves	Du L. et al., 2015
66	Serine	Aqueous	Roots, stems, and leaves	Du L. et al., 2015
67	Asparagine	Aqueous	Roots, stems, and leaves	Du L. et al., 2015
68	Aspartic acid	Aqueous	Roots, stems, and leaves	Du L. et al., 2015
69	Citrulline	Aqueous	Roots, stems, and leaves	Du L. et al., 2015
70	Arginine	Aqueous	Roots, stems, and leaves	Du L. et al., 2015
71	Ornithine	Aqueous	Roots, stems, and leaves	Du L. et al., 2015
**Nucleosides**				
72	Adenosine	EtOH	Flowers	Lai et al., 2006
73	5′-Deoxy-5′-methylsulfinyl adenosine	MeOH	Flowers	Yang et al., 2017
74	Nicotinamide	MeOH	Flowers	Yang et al., 2017
75	Thymidine	Aqueous	Leaves and flowers	Du L. Y. et al., 2015
76	Thymine	Aqueous	Leaves and flowers	Du L. Y.et al., 2015
77	2′-Deoxyuridine	Aqueous	Leaves and flowers	Du L. Y. et al., 2015
78	Uracil	Aqueous	Leaves and flowers	Du L. Y. et al., 2015
79	2′-Deoxyadenosine	Aqueous	Leaves and flowers	Du L. Y. et al., 2015
80	2′-Deoxyinosine	Aqueous	Leaves and flowers	Du L. Y.et al., 2015
81	Adenine	Aqueous	Leaves and flowers	Du L. Y. et al., 2015
82	Inosine	Aqueous	Leaves and flowers	Du L. Y. et al., 2015
83	Cytidine	Aqueous	Leaves and flowers	Du L. Y. et al., 2015
84	Guanine	Aqueous	Leaves and flowers	Du L. Y. et al., 2015
85	2′-Deoxyadenosine-5′-monophosphate	Aqueous	Leaves and flowers	Du L. Y. et al., 2015
86	Cytidine-5′-monophosphate	Aqueous	Leaves and flowers	Du L. Y. et al., 2015
87	Guanosine	EtOH	Flowers	Lai et al., 2006
**Polysaccharides**				
88	Glucose	EtOH	Flowers	Zheng et al., 2016
		EtOH	Stems and leaves	Pan et al., 2018
89	Mannose	EtOH	Flowers	Zheng et al., 2016
		EtOH	Stems and leaves	Pan et al., 2018
90	Galactose	EtOH	Flowers	Zheng et al., 2016
		EtOH	Stems and leaves	Pan et al., 2018
91	Fucose	EtOH	Flowers	Zheng et al., 2016
92	Rhamnose	EtOH	Stems and leaves	Pan et al., 2018
93	Glucuronic acid	EtOH	Stems and leaves	Pan et al., 2018
94	Arabinose	EtOH	Stems and leaves	Pan et al., 2018
**Organic acids**				
95	2,4-Dihydroxybenzoic acid	EtOH	Flowers	Lai et al., 2006
96	4-Hydroxybenzoic acid β-D-glucose ester	EtOH	Flowers	Li et al., 2011
97	Protocatechuic acid	EtOH	Flowers	Li et al., 2011
98	Protocatechuic acid 3-*O*-β-D-glucoside	EtOH	Flowers	Li et al., 2011
99	Caffeic acid	EtOH	Flowers	Chen et al., 2007
100	Palmitic acid	EtOH	Flowers	Chen, 2006
101	Hexacosanoic acid	EtOH	Flowers	Chen, 2006
102	Gallic acid	MeOH	Flowers	Yang et al., 2017
103	3-*O*-caffeoylquinic acid	EtOH	Flowers	Chen, 2006
104	3,5-Di-*O*-caffeoylquinic acid	EtOH	Flowers	Chen, 2006
105	4,5-Di-*O*-caffeoylquinic acid	EtOH	Flowers	Chen, 2006
106	3,4-Di-*O*-caffeoylquinic acid	EtOH	Flowers	Chen, 2006
107	(*E*)-9-octadecenoic acid	MeOH	Flowers	Yang et al., 2017
**Sterols**				
108	Stigmasterol	EtOH	Flowers	Chen et al., 2007
109	α-Spinasterol	EtOH	Flowers	Chen et al., 2007
110	β-Sitosterol	EtOH	Flowers	Chen et al., 2007
		EtOH	Flowers	Lai et al., 2006
111	β-Sitosterol-3-*O*-β-D-glucopyranoside	EtOH	Flowers	Lai et al., 2006
		EtOH	Flowers	Chen et al., 2007
112	β-Daucosterol	EtOH	Flowers	Chen, 2006
**Volatile oils**				
113	Maleic acid	EtOH	Flowers	Lai et al., 2006
114	Tetracosane	EtOH	Flowers	Lai et al., 2006
		EtOH	Flowers	Zhang et al., 2008
115	Hexadecane	EtOH	Flowers	Zhang et al., 2008
116	Heneicosane	EtOH	Flowers	Zhang et al., 2008
117	Octadecane	EtOH	Flowers	Zhang et al., 2008
118	Allyl undecylenate	EtOH	Flowers	Zhang et al., 2008
119	Docosane	EtOH	Flowers	Zhang et al., 2008
120	Hexadecanoic acid	EtOH	Flowers	Zhang et al., 2008
121	Heptatriacontanoic acid	EtOH	Flowers	Lai et al., 2006
122	Tetradecanoic acid	EtOH	Flowers	Zhang et al., 2008
123	Undecanone, 6,10-dimethyl	EtOH	Flowers	Zhang et al., 2008
124	Heptadecane, 2,6,10,15-tetramethyl	EtOH	Flowers	Zhang et al., 2008
125	9,12-Octadecadienoic acid	EtOH	Flowers	Zhang et al., 2008
**Other compounds**				
126	Scopoletin	EtOH	Flowers	Chen, 2006
127	Glycerol monopalmitate	EtOH	Flowers	Lai et al., 2006
128	1-Triacontanol	EtOH	Flowers	Lai et al., 2006

EtOAc, ethyl acetate; EtOH, ethanol; MeOH, methanol.

**Table 2:** Monosaccharide composition, molecular weight, structures, and bioactivities of polysaccharides purified from *Abelmoschus manihot*.

No.	Name	Monosaccharide composition	Molecular weight (Da)	Structures	Bioactivities	Reference
**1**	AMPS-a	Glucose, mannose, galactose, and fucose in a molar ratio of 1.00: 0.91: 2.14: 1.09	8.80 × 10^3^	→6)α-D-Galp-(1→6)α-D-Manp-(1→6)α-D-Galp-(1→ with β-D -Glcp (1→3) α-Fucp-(1→ branching at O-3 of mannose	Antitumor activity	Zheng et al., 2016
**2**	SLAMP-a	Mannose, rhamnose, glucuronic acid, glucose, galactose, and arabinose in a molar ratio of 0.40: ND: ND: 21.93: 1.00: 0.40	ND	ND	Immunomodulatory activity	Pan et al., 2018
**3**	S-SLAMP-a3	Mannose, rhamnose, glucuronic acid, glucose, galactose, and arabinose in a molar ratio of 0.39: ND: ND: 18.91: 1.00: 0.41	1.04 × 10^6^	ND	Immunomodulatory activity	Pan et al., 2018
**4**	SLAMP-c	Mannose, rhamnose, glucuronic acid, glucose, galactose, and arabinose in a molar ratio of 0.19: 1.63: 3.04: 1.15: 1.00: 0.45	4.78 × 10^6^	A triple-helix structure	Immunomodulatory activity	Pan et al., 2018
**5**	SLAMP-d	Mannose, rhamnose, glucuronic acid, glucose, galactose, and arabinose in a molar ratio of 0.54: 1.92: 4.15: 1.64: 1.00: 0.54	2.64 × 10^6^	A triple-helix structure	Immunomodulatory activity	Pan et al., 2018

ND, not detected.

### Flavonoids

Flavonoids, as important secondary metabolites, are widespread throughout the plant kingdom, either in their free form or in the form of glycosides (Liao et al., 2012; He et al., 2017). Since the first study of *A. manihot* flowers in 1981, several flavonoids and their derivatives have been successively purified and identified. Currently, a total of 49 flavonoids (1–49) have been isolated and identified from the flowers of *A. manihot*. Phytochemical studies have indicated that the total flavonoids extracted from the flowers of *A. manihot* (TFAM) are their major pharmacologically active constituents and include seven chemically identified flavone glycosides. TFAM have been proved to exhibit a broad range of pharmacological activities (Yan et al., 2015). The seven main compounds with their contents identified in TFAM using high-performance liquid chromatography (HPLC) are hyperoside (43.2%), hibifolin (27.1%), isoquercitrin (13.7%), quercetin-3′-*O*-β-D-glucopyranoside (8.8%), quercetin-3-*O*-robinobioside (3.8%), myricetin (3.2%), and quercetin (0.2%) (Trendafilova et al., 2011; Xue et al., 2011b; Zhou et al., 2012). In addition, floramanosides A, B, C, D, E, and F (34–39) from *A. manihot* have also been demonstrated to display multiple biological effects *in vivo* and *in vitro* (**Table 1** and **Figure 2**).

### Amino Acids

Most studies have focused on the flowers of *A. manihot*, whereas the compounds from other parts of *A. manihot*, such as the roots, stems, and leaves, have been little reported. Currently, 22 amino acids (50–71), namely phenylalanine (50), leucine (51), isoleucine (52), tryptophan (53), γ-aminobutyric acid (54), methionine (55), valine (56), proline (57), tyrosine (58), alanine (59), hydroxyproline (60), threonine (61), glycine (62), glutamate (63), glutamine (64), lysine (65), serine (66), asparagine (67), aspartic acid (68), citrulline (69), arginine (70), and ornithine (71), have been obtained from the flowers, roots, stems, and leaves of *A. manihot* (Du L. et al., 2015; Liu et al., 2016). Furthermore, it is recognized that amino acids from *A. manihot* contribute to the regulation of whole-body metabolism and play a key role in neurotransmission and lipid transport, as well as being involved in many pharmacological activities (Wang et al., 2010; Zhou et al., 2013; Refaey et al., 2015; Zhang et al., 2015).

### Nucleosides

Nucleotides, nucleosides, and nucleobases are basic components of all cells and form the various types of nucleic acids. They have been proved to be significant biological components related to many physiological processes (Du L. Y. et al., 2015). To date, 16 nucleotides, nucleosides, and nucleobases (72–87) have been isolated and identified from the leaves and flowers of *A. manihot*, namely adenosine (72), 5′-deoxy-5′-methylsulfinyl adenosine (73), nicotinamide (74), thymidine (75), thymine (76), 2′-deoxyuridine (77), uracil (78), 2′-deoxyadenosine (79), 2′-deoxyinosine (80), adenine (81), inosine (82), cytidine (83), guanine (84), 2′-deoxyadenosine-5′-monophosphate (85), cytidine-5′-monophosphate (86), and guanosine (87) (Du L. Y. et al., 2015). These compounds, which have nutraceutical and bioactive properties, might be developed in the future.

### Polysaccharides

Thus far, there have been many studies on flavonoids but few studies on polysaccharides from *A. manihot*. Zheng et al. (2016) isolated a low-molecular-weight polysaccharide, named AMPS-a, from the ethanolic extract of the flowers of *A. manihot*. The results showed that AMPS-a is composed of glucose (88), mannose (89), galactose (90), and fucose (91) in a molar ratio of 1.00 : 0.91 : 2.14 : 1.09 (Zheng et al., 2016). Moreover, three polysaccharides, S-SLAMP-a3, SLAMP-c, and SLAMP-d, were obtained from the stems and leaves of *A. manihot*. These polysaccharides are mainly composed of mannose (89), rhamnose (92), glucuronic acid (93), glucose (88), galactose (90), and arabinose (94) (Pan et al., 2018). In addition, the monosaccharide compositions, molecular weights, structural characteristics, and biological activities of polysaccharides purified from *A. manihot* are listed in **Table 2**.

### Organic Acids

Currently, 13 organic acids (95–107) have been obtained from the flowers of *A. manihot*. Specifically, 2,4-dihydroxybenzoic acid (95), 4-hydroxybenzoic acid β-D-glucose ester (96), protocatechuic acid (97), protocatechuic acid 3-*O*-β-D-glucoside (98), caffeic acid (99), palmitic acid (100), hexacosoic acid (101), gallic acid (102), 3-*O*-caffeoylquinic acid (103), 3,5-di-*O*-caffeoylquinic acid (104), 4,5-di-*O*-caffeoylquinic acid (105), 3,4-di-*O*-caffeoylquinic acid (106), and (*E*)-9-octadecenoic acid (107) were isolated from the ethanolic extract of the flowers of *A. manihot* (Chen, 2006; Lai et al., 2006; Chen et al., 2007; Li et al., 2011). However, the bioactivity of these compounds has not been thoroughly studied and needs further investigation.

### Sterols

To date, only five steroids (108–112) have been purified and characterized from the flowers of *A. manihot*. Stigmasterol (108) has been obtained and identified from the petroleum ether extract of the woody stems of *A. manihot* and identified (Jain et al., 2009). The compounds α-spinasterol (109), β-sitosterol (110), β-sitosterol-3-*O*-β-D-glucopyranoside (111), and β-daucosterol (112) have been isolated and identified from the ethanolic extract of the flowers of *A. manihot* (Chen, 2006; Lai et al., 2006; Chen et al., 2007).

### Volatile Oils

The compositions and contents of volatile oils from the flowers of *A. manihot* have been investigated. Lai et al. (2006) and Zhang et al. (2008) analyzed the major constituents of the volatile oil from the ethanol extract of the flowers of *A. manihot* by gas chromatography–mass spectrometry (MS). The results demonstrated that volatile oil was mainly composed of maleic acid (113, 2.46%), tetracosane (114, 11.02%), hexadecane (115, 1.96%), heneicosane (116, 1.38%), octadecane (117, 1.34%), allyl undecylenate (118, 1.41%), docosane (119, 15.06%), hexadecanoic acid (120, 53.37%), tetradecanoic acid (122, 3.15%), undecanone, 6,10-dimethyl (123, 2.06%), heptadecane, 2,6,10,15-tetramethyl (124, 2.84%), and 9,12-octadecadienoic acid (125, 6.41%) (Lai et al., 2006; Zhang et al., 2008).

### Other Compounds

Scopoletin (126), glycerol monopalmitate (127), and 1-triacontanol (128) have been reported to be present in *A. manihot* (Chen, 2006; Lai et al., 2006). Rao et al. (1990) reported that the leaves of *A. manihot* contained 1.77% lipids, 2.20% proteins, and 1.61% ash, and the content of water reached 88.4%. The lipids consisted of non-polar lipids, glycolipids, and phospholipids (Rao et al., 1990). Jarret et al. (2011) reported that the oil content in the seeds of *A. manihot* reached 16.1–22.0%. Lin et al. (2002) and Wu J. F. et al. (2019) found that a large content of unsaturated fatty acids (91.815%) was present in the seed oil of *A. manihot*, including oleic acid (82.179%), stearic acid (9.195%), linolenic acid (4.756%), palmitoleic acid (2.681%), palmitic acid (0.441%), linoleic acid (0.328%), and other unknown acids (0.748%). Importantly, there are 24 mineral elements (K, Ca, Fe, Mn, Cu, Zn, Mo, etc.) in the seed oil, and the contents of some harmful elements, such as Hg, As, and Cr, were far less than the maximum set by food standards (Lin et al., 2002; Wu J. F. et al., 2019). Therefore, the seeds of *A. manihot* have high nutritional and healthcare value and have vast potential for the development of a series of functional foods.

## Pharmacological Activities

**Figure 3:**
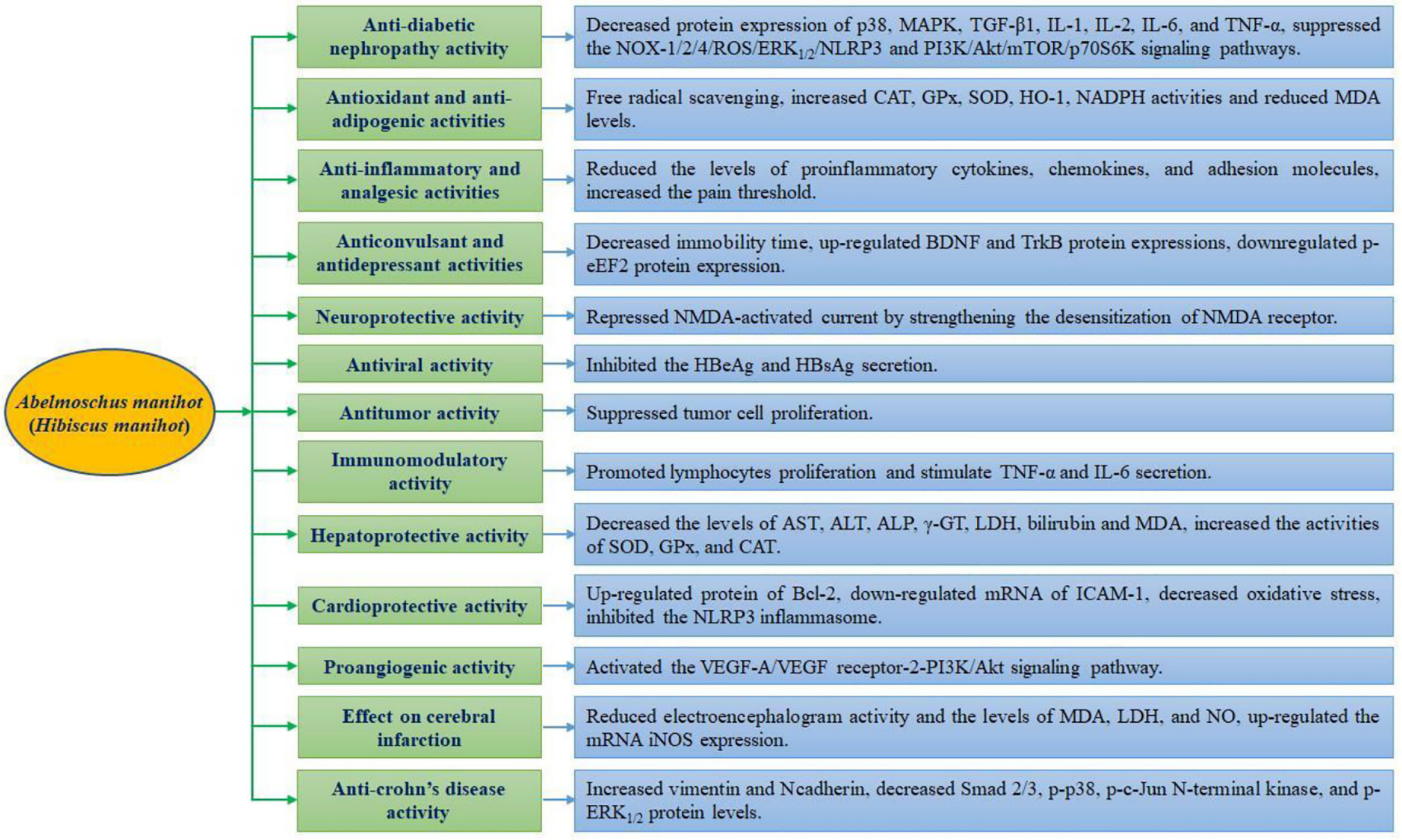
Graphical summary of the pharmacological properties of *Abelmoschus manihot*.

**Figure 4:**
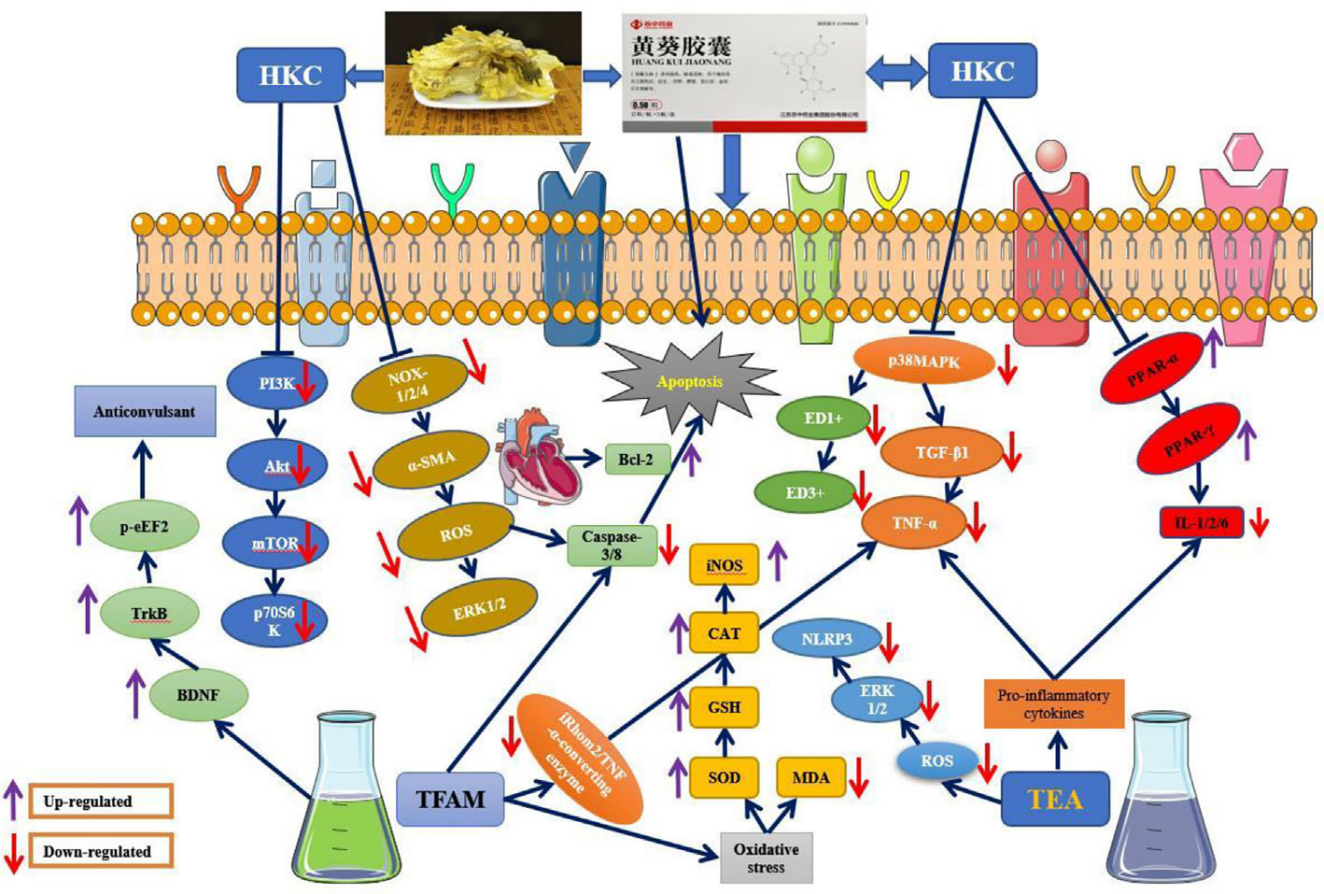
A schematic depiction of the possible mechanisms of action of *Abelmoschus manihot*.

Various pharmacological activities have been reported for the extracts and active compounds from *A. manihot* both *in vitro* and *in vivo* (**Table 3**). Monomer compounds and extracts from different parts of this plant exhibited potent antidiabetic nephropathy, antioxidant, antiadipogenic, anti-inflammatory, analgesic, antiviral, anticonvulsant, antidepressant, antitumor, cardioprotective, antiplatelet, neuroprotective, immunomodulatory, and hepatoprotective activities. The pharmacological properties of the monomer compound and extracts from *A. manihot* as well as a schematic depiction of the possible mechanism of action are presented in **Figures 3** and **4**, respectively.

**Table 3:** Biological properties of extracts or compounds from *Abelmoschus manihot* L. and their possible mechanisms of action observed in the literature.

Biological activity	Extract/compound	Type	Testing subject	Dose and duration	Mechanisms/effects	Reference
Antidiabetic nephropathy activity	HKC	*In vivo*	Doxorubicin-induced nephropathy in rat	0.5 and 2.0 g/kg, i.g., for 28 days	TNF-α, TGF-β1, p-p38MAPK protein expression ↓; infiltrated ED^1+^ and ED^3+^ macrophages ↓	Tu et al., 2013
	HKC	*In vivo*	Unilateral nephrectomy combined STZ-induced DN in rat	0.75 and 2.0 g/kg, i.g., for 56 days	p38MAPK, p-Akt, TGF-β1, TNF-α protein expression ↓; BUN, UA, BW, Alb levels ↓	Mao et al., 2015
	HKC	*In vivo*	STZ-induced DN in rat	75, 135, and 300 mg/kg, i.g., for 84 days	PPAR, CD36, and LPL mRNA ↑; Alb, triglyceride, cholesterol, fat, TNF-α, IL-6, IL-1, and IL-2 levels ↓	Ge et al., 2016
	HKC	*In vitro*	HRMC cells	5 mg/mL	PPAR activities↑; lipoprotein lipase, fatty acid synthase, aP2, and GLUT4 mRNA expression ↑	Ge et al., 2016
	HKC	*In vitro*	HepG2 cells	5 mg/mL	CD36, CPT1, PDK4, and ACO mRNA expression ↑	Ge et al., 2016
	HKC	*In vivo*	Adenine-induced CRF in rats	0.75 g/kg, i.g., for 28 days	Scr, BUN, and UP levels ↓; α-SMA, p-ERK1/2, and NOX-1/2/4 protein expression ↓	Cai et al., 2017a
	HKC	*In vitro*	High glucose-induced EMT in HK-2 cells	100 µM	α-SMA, p-ERK1/2, NOX-1, NOX-2, and NOX-3 protein expression ↓	Cai et al., 2017a
	HKC	*In vivo*	STZ-induced DN in rat	2 g/kg, i.g., for 28 days	p-Akt, p-mTOR, p-p70S6K, and TGF-β1 protein expression ↓	Wu et al., 2018
	Hyperoside	*In vitro*	High glucose-induced in mesangial cells	5 and 15 µg/mL	PI3K, Akt, mTOR, and p70S6K protein expression ↓	Wu et al., 2018
	Hyperoside	*In vivo*	IR-induced AKI in mice	20 mg/kg, i.p.	BUN, Scr, apoptosis, and caspase-3 ↓; DHE fluorescence ↑; OMA1 ↑; OPA1 ↓	Wu L. et al., 2019
	Hyperoside	*In vitro*	CoCl_2_-induced HK-2 cells	50, 100, 150, and 200 µM	Apoptosis, and caspase-3 ↓; ROS levels ↓; OMA1 ↑; OPA1 ↓	Wu L. et al., 2019
	Hyperoside	*In vitro*	Podocyte induced by AGE	50 and 200 µg/mL	Podocyte apoptosis ↓; caspase-3/8 protein expressions ↓	Zhou et al., 2012
	TFAM	*In vivo*	STZ-induced DN rats	0.2 g/kg, i.g., for 168 days	Ratio of urinary microalbumin/creatinine↓; 24 h urinary total protein ↓	Zhou et al., 2012
	TFAM	*In vitro*	HRMC and HK-2 cell lines induced by AGE	20 µM	iRhom2/TACE signaling ↓	Liu et al., 2018
	TFAM	*In vivo*	Unilateral nephrectomy combined STZ-induced DN in rat	0.075, 0.135, and 0.3 g/kg, i.g., for 84 days	Scr, BUN levels ↓; IL-1, IL-2, IL-6, and TNF-α expressions ↓; iRhom2/TACE signaling ↓	Liu et al., 2018
	TEA	*In vivo*	Doxorubicin-induced nephropathy in rat	0.2 g/kg, i.g., for 168 days	Proteinuria, albumin, ROS-ERK1/2-NLRP3 inflammasome protein activation ↓; caspases 3/8 ↓	Li et al., 2019
	TEA	*In vitro*	Doxorubicin-induced NRK-52E cells	100 µg/mL	P38 and ERK1/2 signaling pathway ↓	Li et al., 2019
	Aqueous extract	*In vivo*	High-fat diet and STZ-induced DN mice	0.1 g/kg, i.g., for 35 days	pCr, BUN, and urinary albumin levels ↓	Kim et al., 2018
Antioxidant and antiadipogenic activity	Floramanosides A–F	*In vitro*	DPPH scavenging activity	0–200 µM	DPPH scavenging activity with SC_50_ of 10.1, 6.2, 10.4, 12.5, 24.0, and 25.1 µm, respectively	Zhang et al., 2013
	Floramanosides A–F	*In vitro*	AR inhibitory activity	0–200 μM	AR inhibition activity with IC_50_ of 17.8, 13.7, 7.1, 2.2, and 8.3 µm, respectively	Zhang et al., 2013
	TFAM	*In vitro*	DPPH scavenging activity	25, 50, 100, and 200 µg/mL	DPPH scavenging activity with IC_50_ of 0.288 mg/mL	Li et al., 2016
	TFAM	*In vitro*	3T3-L1 cells	25, 50, 100, and 200 µg/mL	PPARγ and C/EBPα mRNA expression ↓	Li et al., 2016
	TFAM	*In vivo*	D-gal-induced mouse model	40, 80, and 160 mg/kg, i.g., for 42 days	CAT, GPx, SOD, and T-AOC activities ↑; MDA, TNF-α, and IL-1β level ↓; Nrf2, HO-1 and NQO1 protein expression ↑; GPx, SOD, and CAT mRNA expression ↑	Qiu et al., 2017
Anti-inflammatory and analgesic activity	Petroleum ether and methanol extracts	*In vivo*	Carrageenan and histamine-induced paw edema model	100, 200, and 400 mg/kg, i.g.	Edema volume ↓	Jain et al., 2009; Jain and Bari, 2010
	Ethanol extracts	*In vivo*	DSS-induced colitis in mice	0.25, 0.5, and 1.0 mg/g, i.g., for 6 days	IL-1β, IL-6, IL-17, IL-22, TNF-α, CXCL1, CXCL2, CXCL9, CXCL10, CCL-2, Madcam, P-selectin, and E-selectin mRNA expression ↓	Zhang et al., 2019
	TFAM	*In vivo*	Acetic acid writhing test in mouse model	5, 10, and 20 mg/kg, i.p.	Inhibition rate of 57.53%, 42.81%, and 57.19%, respectively	Fan et al., 2003
	TFAM	*In vivo*	Formalin pain test, and KCl test in mouse model	140 and 280 mg/kg, i.g.	Phase I and phase II in the formalin pain ↓	Fan et al., 2003
	Petroleum ether and methanol extracts	*In vivo*	Tail immersion and hot plate model in mice	100, 200, and 400 mg/kg, i.g.	Pain threshold ↓	Pritam et al., 2011
Anticonvulsant and antidepressant activity	Compounds (**19**) and (**25**)	*In vivo*	TST- and FST-induced mouse model	15, 30, and 60 mg/kg, i.g., for 1 day	TST, FST ↓; BDNF, TrkB ↑; p-eEF2 ↓	Cai et al., 2017b
	TFAM	*In vivo*	PSD in rats	25, 50 and 100 mg/kg, i.g., for 24 days	SOD, GSH-Px activities ↑; MDA ↓	Hao et al., 2007
	TFAM	*In vivo*	PSD injury in rats	40, 80, and 160 mg/kg, i.g., for 24 days	SOD, GSH-Px activities ↑; MDA ↓; BDNF and CREB mRNA and protein expressions ↑	Liu et al., 2009
	Ethanol extract	*In vivo*	PTZ-induced clonic convulsions and mortality in mice	100 and 200 mg/kg, i.g.	Survival time↑; mortality rate ↓	Guo et al., 2011
Neuroprotective activity	TFAM	*In vivo*	Acute incomplete cerebral ischemia in rats	50 and 100 mg/kg, i.g., for 3 days	Incidence of brain edema ↓	Gao et al., 2003
	TFAM	*In vitro*	Cultured rat hippocampal neurons	0.2 mg/mL	NMDA receptor desensitization ↑	Cheng et al., 2006
Antiviral activity	Hyperoside	*In vitro*	HepG2.2.15 cells	0.05 g/L	Inhibition rates of HBeAg and HBsAg were 86.41% and 82.27%, respectively	Wu et al., 2007
	Hyperoside	*In vivo*	DHBV infection duckling model	0.05 and 0.1 g/kg, i.g., for 10 days	DHBV-DNA levels ↓	Wu et al., 2007
Antitumor activity	Polysaccharide	*In vitro*	SMMC-7721and HepG2; MGC-803 and MKN-45	50–400 μg/mL	Antiproliferation	Zheng et al., 2016
	HKC	*In vivo*	Multiple myeloma-prone mouse model	3.75 g/kg, i.g., 3 times a week	Survival rate ↑	Hou et al., 2020
Immunomodulatory activity	Polysaccharide	*In vitro*	RAW264.7 cells	50, 100, and 200 μg/mL	TNF-α, IL-6 secretion ↑; spleen lymphocyte proliferation ↑	Pan et al., 2018
Hepatoprotective activity	TFAM	*In vivo*	CCl_4_-induced acute liver damage in mice	125, 250, and 500 mg/kg, i.g., for 7 days	MDA, ALT, AST, ALP, and γ-GT levels ↓; GSH, GPx, CAT, and GST activities ↑	Ai et al., 2013
	TFAM	*In vitro*	CCl_4_-exposed hepatocytes	9, 18, 36, and 72 mg/L	ALT, AST, and ALP level ↓	Ai et al., 2013
	TFAM	*In vivo*	ANIT-induced liver injury in rats	125, 250, and 500 mg/kg, i.g., for 9 days	ALT, AST, LDH, ALP, GGT, TBIL, DBIL, and TBA levels ↓; MDA, TNF-α, and NO contents ↓; SOD, GSH, and GST activities ↑; BSEP, MRP2, and NTCP mRNA expression ↑	Yan et al., 2015
Cardioprotective activity	TFAM	*In vivo*	Myocardial ischemia–reperfusion injury in rats	100 and 200 mg/kg, i.p.	MDA, CPK, LDH ↓; SOD activity ↑; apoptotic cells ↓; Bcl-2 expression ↑	Li et al., 2006
	TFAM	*In vivo*	Myocardial ischemia–reperfusion in rabbits	4, 8, and 16 mg/kg, i.v.	SOD, GSH-Px activities ↑; MDA level ↓; ICAM-1 mRNA expression ↓	Fan et al., 2006
	TFAM	*In vivo*	Myocardial ischemia–reperfusion in rats	40 and 80 mg/kg, i.g.	CK, LDH, IL-6, IL-1β, and TNF-α levels ↓; SOD activity ↑; MDA content ↓	Lv et al., 2017
Proangiogenic activity	TFAM	*In vitro*	HUVECs	5, 10, and 20 μg/mL	Induced the HUVECs proliferation, migration, invasion, and tube formation; VEGF-A, VEGFR2, PI3K, and Akt protein expressions ↑	Zhu et al., 2018
	TFAM	*In vivo*	Chick CAM model	0, 5, 10, and 20 μg/mL	Promoted the formation of blood vessels	Zhu et al., 2018
Effect on cerebral infarction	TFAM	*In vivo*	Nitrogen anoxia model in mice	30, 60, and 120 mg/kg, i.v.	Survive time ↑; MDA content ↓	Guo and Chen, 2002
	TFAM	*In vivo*	Cerebral ischemia–reperfusion in rabbits	12, 24, and 48 mg/kg, i.v.	EEG, MDA, and LDH levels ↓	Guo and Chen, 2002
	TFAM	*In vivo*	MCA rat model	25, 50, and 100 mg/kg, i.g.	Cerebral infarction weight ↓; LDH and NO ↑	Gao et al., 2002
	TFAM	*In vivo*	MCA rat model	20, 40, 80, and 160 mg/kg, i.g.	Cerebral infarction size ↓; LDH, MDA ↓; iNOS mRNA expression ↑	Wen and Chen, 2007
Anti-Crohn’s disease activity	TFAM	*In vitro*	TGF-β1-induced EMT in IEC-6 cells	5, 10, and 15 µg/mL	E­cadherin and ZO­1 mRNA and protein expressions ↑; vimentin, N­cadherin, Smad 2/3, p­p38, p­JNK and p­ERK_1/2_ mRNA and protein ↓	Yang B. L. et al., 2018
Effect on bone loss	*A. manihot* leaves	*In vivo*	Ovariectomized rats	15% of leaves of in the diet	BMD and BMC ↑	Puel et al., 2005
Antiplatelet activity	TFAM	*In vivo*	Rat models induced by artery–vein bypassing thrombus formation	25, 50, and 100 mg/kg, i.g., for 3 days	Thrombus weight ↓	Guo et al., 2005
	TFAM	*In vitro*	Collagen-induced platelet aggregation in rabbits	0.025, 0.05, 0.10 mg/mL	Resting ↓; free intracellular calcium concentration ↑	Guo et al., 2005
Other activity	Hyperoside	*In vivo*	Ethylene glycol-fed rats	20 mg/kg, i.g., for 21 days	Crystal deposit numbers ↓; SOD, CAT activities ↑	Zhu et al., 2014

AKI, acute kidney injury; AR, aldose reductase; CAM, chorioallantoic membrane; CRF, chronic renal failure; EMT, epithelial-mesenchymal transition; MCA, middle cerebral artery; PSD, poststroke depression; ACO, acyl-CoA oxidase; AGE, dvanced glycation end-products; Akt, serinethreonine kinase; Alb, albumin; ALP, alkaline phosphatase; ALT, alanine aminotransferase; ANIT, α-naphthylisothiocyanate; aP2, adipose fatty acidbinding protein; AST, aspartate aminotransferase; BDNF, brain-derived neurotrophic factor; BMC, bone mineral content; BMD, bone mineral density; BSEP, bile salt export pump; BUN, blood urea nitrogen; BW, body weight; CAT, catalase; CK, creatinine kinase; CPK, creatine phosphokinase; CREB, cAMP-response element-binding protein; DBIL, direct bilirubin; DHBV, duck hepatitis B virus; DHBV-DNA, duck hepatitis B virus-DNA; DHE, dihydroethidium; DN, diabetic nephropathy; DPPH, 1,1-diphenyl-2-picrylhydrazyl; DSS, dextran sulfate sodium; ED, macrophage; EEG, electroencephalography; EMT, pithelial-mesenchymal transition; FST, forced swimming test; GGT, gamma-glutamyltransferase; GLUT4, glucose transporter-4; GPx, glutathione peroxidase; GSH, glutathione; GSH-Px, glutathione-Px; GST, glutathione transferase; HBeAg, hepatitis Be antigen; HBsAg, hepatitis B surface antigen; HKC, Huangkui capsule; HO-1, heme oxygenase-1; HRMC, human renal mesangial cells; HUVEC, human umbilical vein endothelial cells; i.g., intragastrically; i.p., intraperitoneally; IC50, 50% inhibitory concentration; ICAM, intercellular cell adhesion molecule; IL, interleukin; iNOS, nitric oxide synthase; IR, ischemia/reperfusion; LDH, lactate dehydrogenase; LPL, lipoprotein lipase; MAPK, mitogen-activated protein kinase; MDA, malondialdehyde; MRP2, multidrug resistance-associated protein 2; NMDA, N-methyl-D-aspartic acid; NQO1, NAD(P)H: quinoneoxidoreductase NQO1:EC1.6.99.2; Nrf2, nuclear factor(erythroid-derived2)-like 2 protein; NTCP, Na+-taurocholate cotransporting polypeptide; OMA1, metalloendopeptidase OMA1; OPA1, optic atrophy 1; pCr, plasma creatinine; PI3K, phosphatidylinositol-3-kinase; PPAR, peroxisome proliferator-activated receptors; PTZ, pentylenetetrazole; ROS, reactive oxygen species; SC50, 50% scavenging concentration; Scr, serum creatinine; SMA, a-smooth muscle actin; SOD, superoxide dismutase; STZ, streptozotocin; TACE, tumor necrosis factor-a converting enzyme; T-AOC, total antioxidant capacity; TBA, total bile acid; TBIL, total bilirubin; TEA, total extracts of A. manihot flower; TFAM, total flavones of A. manihot; TGF, transforming growth factor-β1; TNF, tumor necrosis factor; TrkB, tyrosine receptor kinase; TST, tail suspension test; UA, serum uric acid; UP, urine protein; VEGF-A, vascular endothelial growth factor-A; VEGFR2, VEGF receptor-2; γ-GT, gamma glutamyltransferase.

### Antidiabetic Nephropathy Activity

DN is a common complication of diabetes that has become a serious threat to human health and life and is expected to become the commonest cause of end-stage renal disease and cardiovascular events (Balakumar et al., 2012; Lv et al., 2015; Ge et al., 2016). In a rat model of unilateral nephrectomy and doxorubicin-induced nephropathy, HKC (at doses of 0.5 and 2.0 g/kg by intragastric [i.g.] administration for 28 days) notably improved the general status of rats; alleviated renal histological changes, proteinuria, albuminuria, and glomerulosclerosis; decreased the infiltration of ED^1+^ and ED^3+^ macrophages into the glomeruli; and inhibited the protein expression of tumor necrosis factor (TNF)-α in the kidney (Tu et al., 2013; Zhao et al., 2019). Moreover, mechanism studies showed that HKC significantly downregulated the protein expression of transforming growth factor (TGF)-β1 and p38-mitogen-activated protein kinase (MAPK) by suppressing the p38/MAPK signaling pathway in a rat model of doxorubicin-induced nephropathy (Tu et al., 2013; Zhao et al., 2019). In a rat model of DN induced by unilateral nephrectomy and streptozotocin (STZ) injections in comparison with α-lipoic acid, HKC at doses of 0.75 and 2.0 g/kg (i.g.) for 56 days significantly reduced urinary albumin levels; improved renal function by decreasing the blood urea nitrogen (BUN) and serum uric acid levels; alleviated kidney fibrosis by reducing the number of cells and the amount of extracellular matrix in the glomeruli; and reversed increases in markers of oxidative stress, such as malondialdehyde (MDA), 8-hydroxy-2′-deoxyguanosine, total superoxide dismutase (SOD), and nicotinamide adenine dinucleotide phosphate oxidase-4 (Mao et al., 2015). Further mechanism studies proved that HKC simultaneously decreased the protein expression of p-p38MAPK, p-Akt, TGF-β1, and TNF-α by inhibiting the p38MAPK and Akt signaling pathways in the kidney in a rat model of DN (Mao et al., 2015). Later, in *in vitro* and *in vivo* studies, HKC (at doses of 75, 135, and 300 mg/kg [i.g.] for 84 days) increased the mRNA expression of peroxisome proliferator-activated receptor (PPAR)-α and PPAR-γ in the livers and kidneys of rats with DN. HKC also increased serum albumin levels and decreased levels of serum triglycerides, cholesterol, and total fats in a dose-dependent manner in the livers of rats with DN in comparison with irbesartan (Ge et al., 2016). Moreover, HKC decreased the expression of interleukin (IL)-1, IL-2, IL-6, and TNF-α by suppressing the inflammatory reaction in the kidneys of rats with DN. Strikingly, HKC alleviated endoplasmic reticulum stress and decreased the activation of c-Jun NH_2_-terminal kinase in the livers and kidneys of rats with DN and subsequently reduced renal injury (Ge et al., 2016). The results of the above studies demonstrate that HKC might be a candidate agent for treating DN in humans.

HKC (at a dose of 0.75 g/kg [i.g.] for 28 days) significantly decreased the levels of BUN, serum creatinine, and urine protein in plasma, and molecular mechanisms demonstrated that HKC notably downregulated the protein expression of NADPH oxidase (NOX)-1, NOX-2, NOX-4, α-smooth muscle actin (α-SMA), and p-extracellular signal-regulated kinase (ERK)_1/2_ by inhibiting the NADPH oxidase/ROS/ERK signaling pathways in renal tissue in rats with chronic renal failure induced by adenine *in vivo* (Cai et al., 2017a). Phytochemical investigations showed the main bioactive components of HKC; namely, quercetin, quercetin-3′-*O*-glucoside, isoquercitrin, and hyperoside. In particular, gossypetin-8-*O*-β-D-glucuronide, at a concentration of 100 µM, significantly inhibited the protein expression of α-smooth muscle actin, p-ERK1/2, NOX-1, NOX-2, and NOX-4 in HK-2 cells induced by high glucose levels in the same way as the NOX inhibitor diphenyleneiodonium (Cai et al., 2017a). These *in vivo* and *in vitro* results suggest that HKC and its major flavonoid components protect against tubulointerstitial fibrosis in rats with chronic renal failure by suppressing the NOX/reactive oxygen species (ROS)/ERK signaling pathway. Furthermore, HKC (at a dose of 2 g/kg [i.g.] for 28 days) dramatically reduced urinary microalbumin levels, increased body weight and serum albumin levels, improved renal morphology and kidney weight, reduced the kidney hypertrophy index, alleviated glomerular hypertrophy, decreased the expression of α-smooth muscle actin and proliferating nuclear cell antigen, and decreased thickening of the glomerular basement membrane. The mechanism explored demonstrated that HKC downregulated the protein expression of p-p70S6K, p-mammalian target of rapamycin (mTOR), TGF-β1, and p-Akt by repressing the phosphoinositide-3-kinase (PI3K)/Akt/mTOR/p70S6K signaling pathway in the kidneys of rats in a model of early DN (Wu et al., 2018). In agreement with the results of the *in vivo* study, hyperoside, which is a bioactive component of HKC, at concentrations of 5 and 15 µg/mL significantly downregulated the protein expression of p-Akt, p-mTOR, p-PI3K, and p-p70S6K in murine mesangial cells induced by high glucose levels *in vitro* (Wu et al., 2018), which suggested that HKC safely and efficiently alleviates early pathological changes in the glomeruli in DN by inhibiting the PI3K/Akt/mTOR/p70S6K signaling pathway *in vivo* and *in vitro* and provides reliable evidence of the prevention of early DN.

In an *in vitro* study, TFAM at a concentration of 20 μM suppressed the activation of iRhom2/TNF-α-converting enzyme in human renal mesangial cells and HK-2 cells induced by advanced glycation end products (Liu et al., 2018). Similarly, the results of an *in vivo* study demonstrated that TFAM (at doses of 75, 135, and 300 mg/kg [i.g.] for 84 days) notably decreased the levels of serum creatinine and BUN by downregulating the expression of IL-1, IL-2, IL-6, and TNF-α in a dose-dependent manner by suppressing the activation of the iRhom2/TNF-α-converting enzyme signaling pathway in a rat model of DN induced by unilateral nephrectomy and STZ injections in comparison with 4-phenylbutanoic acid (2.5 mg/kg) as a positive control (Liu et al., 2018). Kim et al. (2018) established a mouse model of DN induced by a high-fat diet and STZ after unilateral nephrectomy. The results showed that extracts of *A. manihot* (100 mg/kg [i.g.] for 35 days) markedly reduced the levels of plasma creatinine, BUN, and urinary albumin. Moreover, the kidney/body weight ratio also increased in mice with DN in comparison with control mice (Kim et al., 2018).

The total extract of *A. manihot* flowers (TEA) at a concentration of 100 μg/mL reduced doxorubicin-induced changes in cellular histology, decreases in cell viability, and apoptosis in NRK-52E cells by suppressing protein oxidation and the p38 and ERK_1/2_ signaling pathways *in vitro* (Li et al., 2019). Similarly, the results of an *in vivo* study proved that TEA at a dose of 1.5 g/kg (i.g.) significantly reduced proteinuria and low serum albumin levels, alleviated lesions of the renal tubules, and inhibited the expression of ROS, ERK_1/2_, and NLRP3 inflammasome proteins in the renal tubules of rats with doxorubicin-induced nephropathy in comparison with a doxorubicin model group (Li et al., 2019), which indicates that TEA protects renal tubular cells against doxorubicin-induced injury by suppressing the ROS-ERK_1/2_-NLRP3 inflammasome signaling pathway both *in vitro* and *in vivo*.

In addition, oral administration of TFAM at a dose of 200 mg/kg for 24 weeks significantly decreased the ratio of urinary microalbumin to creatinine and 24 h urinary total protein and reduced the apoptosis of glomerular cells in a rat model of STZ-induced DN (Zhou et al., 2012). Moreover, one of the major active constituents of TFAM, namely hyperoside, at concentrations of 50 and 200 µg/mL, significantly decreased the apoptosis of podocytes induced by advanced glycation end products by suppressing the protein expression of caspase-3 and caspase-8 (Zhou et al., 2012). Simultaneously, in a mouse model of ischemia–reperfusion-induced acute kidney injury, pretreatment with hyperoside (20 mg/kg by intraperitoneal [i.p.] administration) reduced the extent of injury to the renal tubules, decreased the levels of BUN and serum creatinine, reduced the apoptosis of tubular cells, and suppressed the production of ROS and the expression of caspase-3 in the kidneys (Wu L. et al., 2019). Hyperoside also suppressed mitochondrial fission by suppressing OMA1-mediated proteolysis of optic atrophy 1. In agreement with the findings of the *in vivo* study, hyperoside (at concentrations of 50, 100, 150, and 200 μM) prevented cobalt chloride (CoCl_2_)-induced apoptosis and inhibited the cleavage of caspase-3 in HK-2 cells by modulating the OMA1–optic atrophy 1 axis in an *in vitro* study (Wu L. et al., 2019). According to these findings, hyperoside exerts its renoprotective activity partly because of its antiapoptotic and antioxidant activities and might have potential as a novel therapeutic agent for the treatment of acute kidney injury.

### Antioxidant and AntiAdipogenic Activities

Oxidative stress has been implicated in the pathophysiology of various ocular diseases, including cerebral ischemia, atherosclerosis, inflammation, diabetes, and cancer (Ghanem et al., 2010; Zhang et al., 2013). Zhang et al. (2013) found that floramanosides A, B, C, D, E, and F, isolated from the flowers of *A. manihot*, have strong antioxidative and radical scavenging activities against DPPH with 50% scavenging concentrations (SC_50_) of 10.1, 6.2, 10.4, 12.5, 24.0, and 25.1 µM, respectively. Moreover, floramanosides A, B, C, D, and E exhibited significant inhibitory activity against aldose reductase, with half maximal inhibitory concentration (IC_50_) values of 17.8, 13.7, 7.1, 2.2, and 8.3 µM, respectively (Zhang et al., 2013). In addition, hyperoside exhibited notable DPPH scavenging activity, with an IC_50_ value of 0.288 mg/mL. Furthermore, hyperoside at a concentration of 100 µg/mL exhibited significant antiadipogenic activity and downregulated the mRNA expression of PPAR-γ and CCAAT/enhancer binding protein-α in a 3T3-L1 cell line (Li et al., 2016). These results indicate that *A. manihot* flowers may have potential as an agent for treating oxidative stress-related diseases.

In a D-galactose-induced mouse model, TFAM at doses of 40, 80, and 160 mg/kg (i.g.) for 42 consecutive days significantly boosted the activities of catalase (CAT), glutathione peroxidase (GPx), and SOD; increased total antioxidant capacity; and decreased the content of MDA in the liver with a dose-dependent manner when compared with ascorbic acid (80 mg/kg) as a positive control (Qiu et al., 2017). Furthermore, after treatment with TFAM in Nrf2-mediated antioxidant responses, the protein expression of Nrf2, heme oxygenase-1, and NAD(P)H quinone oxidoreductase-1 as well as the mRNA expression of GPx, SOD, and CAT dramatically increased, whereas the TNF-α and IL-1β expression significantly decreased (Qiu et al., 2017). However, investigations into the antioxidant activity of TFAM and the precise mechanism of action involved *in vivo* are very limited.

### Anti-Inflammatory and Analgesic Activities

Petroleum ether and methanolic extracts of the woody stems of *A. manihot* at doses of 100, 200, and 400 mg/kg (i.g.) displayed notable anti-inflammatory activity in the rat model of carrageenan- and histamine-induced paw edema by dose-dependently reducing the percentage increase in the volume of edema in comparison with the standard drug diclofenac sodium (10 mg/kg) as a positive control (Jain et al., 2009; Jain and Bari, 2010). The ethanolic extracts of *A. manihot* at doses of 0.25, 0.5, and 1.0 mg/g (i.g.) for 6 days significantly alleviated dextran sulfate sodium-induced colitis in mice by regulating the composition of the gut microbiota, boosted microbial diversity, and increased the abundance and levels of gut microbiota that produce straight-chain fatty acids, especially butyric acid and acetic acid (Zhang et al., 2019). In addition, treatment with ethanolic extracts of *A. manihot* dramatically decreased the mRNA levels of proinflammatory cytokines such as IL-22, IL-17, IL-6, IL-1β, and TNF-α, chemokines such as CXCL-1, CXCL-2, CXCL-9, CXCL-10, and CCL-2, and adhesion molecules such as mucosal address in cell adhesion molecule, P-selectin, and E-selectin (Zhang et al., 2019), which mainly acted *via* the PPAR-γ signaling pathway and resulted in an increase in the generation of Treg cells and inhibition of the development of Th17 cells.

Petroleum ether and methanolic extracts of *A. manihot* leaves (at doses of 100, 200, and 400 mg/kg, i.g.) exhibited significant analgesic activity in mouse tail immersion and hot plate models by markedly increasing the pain threshold in a dose-dependent manner in comparison with pentazocine (10 mg/kg) as a control (Pritam et al., 2011). TFAM has displayed remarkable analgesic effects in models of acetic acid-induced writhing, formalin-induced pain, and KCl-induced reactions. TFAM at doses of 5, 10, and 20 mg/kg (i.p.) significantly suppressed acetic acid-induced writhing in mice, with inhibition rates of 57.53%, 42.81%, and 57.19%, respectively. TFAM at doses of 140 and 280 mg/kg (i.g.) markedly reduced acetic acid-induced writhing in mice, with inhibition rates of 62.16% and 42.34%, respectively (Fan et al., 2003). Moreover, TFAM markedly alleviated the reaction induced by KCl in rabbits after intra-arterial injection at 200 mg/kg (Fan et al., 2003). Advanced studies should be carried out to further investigate the related biochemical pathways and develop safe and effective analgesic agents.

### Anticonvulsant and Antidepressant Activities

Depression is characterized by a disturbance of mood associated with alterations in behavior, energy, and appetite and is a widespread incapacitating psychiatric disorder (Nemeroff, 2007). In pentylenetetrazole-induced clonic convulsions and associated mortality in mice, Guo et al. (2011) found that oral administration of ethanolic extract of *A. manihot* flowers at a dose of 200 mg/kg (i.g.) greatly prolonged the time to death and decreased the mortality rate and the immobility time, but it had no effect on the latency time in comparison with fluoxetine (20 mg/kg, i.g.) (Guo et al., 2011). In addition, TFAM (at doses of 25, 50, and 100 mg/kg [i.g.] for 24 days) significantly increased the crossing and rearing scores in an open-field test; suppressed increases in the viscosity of whole blood, as well as plasma, at high, moderate, and low shear rates; decreased the deformation of erythrocytes; enhanced the activities of SOD and GPx; and decreased the MDA content in the brains of rats with poststroke depression in comparison with fluoxetine (1.8 mg/kg) (Hao et al., 2007). Most importantly, *A. manihot* potentiated the antimobility effect of fluoxetine and fluoxetine hydrochloride (conventional antidepressants), which confirmed the therapeutic effect of this plant against depression.

TFAM at doses of 40, 80, and 160 mg/kg (i.g.) for 24 days dramatically alleviated escape-directed behavioral impairment in mice induced by poststroke depression, significantly decreased the content of MDA, and increased the activities of SOD and GPx in comparison with fluoxetine (2.5 mg/kg). It also alleviated poststroke depression-induced neuronal death and loss by upregulating the mRNA and protein expressions of brain-derived neurotrophic factor and CREB, which suggests that TFAM has a protective effect against poststroke depression-induced injury in mice (Liu et al., 2009). Further studies demonstrated that the active compounds gossypetin-8-*O*-β-D-glucuronide and quercetin-3’-*O*-glucoside (at doses of 15, 30, and 60 mg/kg, i.g.) of TFAM dramatically shortened the immobility time in the tail suspension and forced-swimming tests, notably increased the expression of brain-derived neurotrophic factor (BDNF) and tropomyosin receptor kinase-B (TrkB), and decreased the expression of p-eukaryotic elongation factor-2 (p-eEF2) in the hippocampus in comparison with ketamine (10 mg/kg) as a positive control (Cai et al., 2017b). These results will provide new insights into the development of antidepressant agents for the treatment of major depressive disorder.

### Neuroprotective Activity

TFAM at doses of 50 and 100 mg/kg (i.g.) for 3 days significantly reduced the incidence of brain edema and alleviated pathological changes in brain tissues in rats with acute incomplete cerebral ischemia in comparison with *Ginkgo biloba* extract (50 mg/kg) as a positive control (Gao et al., 2003). In cultured rat hippocampal neurons, TFAM quickly and reversibly suppressed the *N*-methyl-D-aspartate (NMDA)-activated current in a concentration-dependent manner with an IC_50_ value of 0.46 mg/mL, and also non-competitively suppressed the NMDA-activated current by strengthening the desensitization of NMDA receptors in the presence of TFAM (0.2 mg/mL), which suggests that TFAM exerts a neuroprotective function by inhibiting the NMDA receptor response (Cheng et al., 2006).

### Antiviral Activity

Wu et al. (2007) found that hyperoside at a concentration of 0.05 g/L notably suppressed the secretion of HBeAg and HBsAg in HepG2.2.15 human hepatoma cells with inhibition rates of 86.41% and 82.27%, respectively (Wu et al., 2007). In addition, hyperoside (at doses of 0.05 and 0.1 g/kg [i.g.[for 10 days) significantly reduced the DNA levels of duck HBV in a duckling model of duck HBV infection, and the decreases in peak viremia reached 60.79% on day 10 and 69.78% on day 13, respectively. These results suggest that hyperoside is an effective inhibitor of the secretion of HBsAg and HBeAg in HepG2.2.15 cells and decreases DNA levels of duck HBV in a duck model of HBV infection (Wu et al., 2007). Further studies should be performed to elucidate the molecular mechanism of hyperoside for HBV.

### Antitumor Activity

AMPS-a, a polysaccharide purified from the ethanolic extract of *A. manihot* flowers, at concentrations ranging from 50 to 400 μg/mL, remarkably suppressed the proliferation of human hepatic carcinoma cells (SMMC-7721 and HepG2) and gastric cancer cells (MGC-803 and MKN-45) (Zheng et al., 2016). However, more evidence is needed to obtain detailed structural information as well as the mechanism of action of AMPS-a and explore it as a potential antitumor agent. In a recent study, Hou et al. (2020) found that HKC, at 3.75 g/kg/day, prominently prolonged the survival rate of a multiple myeloma-prone mouse model. Further phytochemical investigations proved that four bioactive ingredients from HKC, namely hyperoside (28), cannabiscitrin (29), 8-(2′′-pyrrolidione-5-yl)-quercetin (48), and 3-*O*-kaempferol-3-*O*-acetyl-6-*O*-(p-coumaroyl)-β-D-glucopyranoside (49), at concentrations of 0.05 and 5 μM, notably promoted the differentiation of murine pre-osteoblast MC3T3-E1 cells (Hou et al., 2020). Furthermore, compound (49) suppressed the proliferation of multiple myeloma ARP1 and H929 cells and induced cell cycle arrest at G_0_/G_1_ phase, which may be related to inhibition of the β-catenin protein, upregulation of the expressions of IL-6 and TNF-α, as well as activation of mature TGF-β1 (Hou et al., 2020). The results from this study indicated that HKC exerts protective effects and may serve as a promising anti-multiple myeloma drug.

### Immunomodulatory Activity

Four polysaccharides, SLAMP-a, S-SLAMP-a3, SLAMP-c, and SLAMP-d, isolated from the stems and leaves of *A. manihot*, at concentrations of 50, 100, and 200 μg/mL, exhibited significant immunomodulatory activity by promoting the proliferation of spleen lymphocytes and stimulating the secretion of TNF-α and IL-6 in RAW264.7 cells. Especially, the sulfated derivatives S-SLAMP-a3 exhibited the highest immunomodulatory activities and the proliferation rate reached 1.47 (Pan et al., 2018), which indicates that sulfated derivatives of polysaccharides might be promising candidates for the development of antitumor drugs. This also provides new insights into the utilization of enormous amounts of discarded resources to avoid extensive waste, as well as environmental pollution.

### Hepatoprotective Activity

The antihepatotoxic activity of crude extracts of *A. manihot* (TFAM) has been demonstrated both *in vitro* and *in vivo*. In acute liver damage induced by CCl_4_ (0.12%, v/v, dissolved in olive oil, 10 mL/kg body weight) in mice, oral administration of TFAM at doses of 125, 250, and 500 mg/kg daily for 7 days significantly decreased the contents of AST, ALT, ALP, and γ-GT in serum in comparison with biphenyldicarboxylate (150 mg/kg) as a positive control (Ai et al., 2013). In addition, TFAM decreased the MDA content and increased the activities of SOD, GPx, CAT, and glutathione-S-transferase in the liver in a dose-dependent manner in comparison with a control group treated with CCl_4_. Histological analyses of the liver also showed that TFAM reduced the extent of liver lesions induced by CCl_4_ (Ai et al., 2013). Moreover, treatment with different concentrations of TFAM (i.e., 9, 18, 36, and 72 mg/L) significantly decreased the levels of ALT, AST, and ALP in the medium in hepatocytes exposed to CCl_4_ in an *in vitro* study (Ai et al., 2013). Similarly, TFAM (at doses of 125, 250, and 500 mg/kg [i.g.] for 9 consecutive days) in a dose-dependent manner notably reduced the levels of AST, AST, LDH, γ-GT, total bilirubin, direct bilirubin, and total bile acids in serum, decreased the contents of MDA, TNF-α, and NO, and increased the activities of SOD, glutathione, and glutathione-S-transferase in liver tissue in cholestatic liver injury induced by α-naphthylisothiocyanate in rats (Yan et al., 2015). Further studies demonstrated that pretreatment with TFAM significantly upregulated the protein and mRNA expression of the bile salt export pump multidrug resistance-associated protein-2 and Na^+^-taurocholate-cotransporting polypeptide in liver injury with cholestasis induced by α-naphthylisothiocyanate (Yan et al., 2015).

### Cardioprotective Activity

TFAM at doses of 100 and 200 mg/kg (i.p.) notably reduced the content of MDA in the myocardium and the production of creatine phosphokinase in serum, increased the activity of SOD and Bcl-2 expression, and decreased the number of apoptotic cells and LDH release in rat myocardium injured by ischemia–reperfusion when compared with nifedipine (2 mg/kg) as a positive control (Li et al., 2006). Intravenous (i.v.) administration of TFAM at doses of 4, 8, and 16 mg/kg significantly alleviated myocardial injury induced by ischemia–reperfusion in rabbits, increased the activities of SOD and GPx, and reduced the level of MDA in plasma. Moreover, the mRNA expression of intercellular adhesion molecule-1 (ICAM-1) in the myocardium was significantly downregulated in rabbits in comparison with verapamil (0.8 mg/kg) as a positive control (Fan et al., 2006). TFAM at doses of 40 and 80 mg/kg (i.p.) exhibited notable cardioprotective activity by decreasing the levels of creatinine kinase, LDH, IL-6, IL-1β, and TNF-α in serum, increasing the activity of SOD, and reducing the MDA content in a rat model of myocardial ischemia–reperfusion. In addition, TFAM alleviated myocardial injury induced by ischemia–reperfusion in rats by suppressing the NLRP3 inflammasome (Lv et al., 2017). Taken together, these results suggest that TFAM might be a candidate drug for treating cardiovascular diseases.

### Proangiogenic Activity

TFAM at concentrations of 5, 10, and 20 μg/mL significantly induced the proliferation, migration, invasion, and formation of human umbilical vein endothelial cells by boosting the phosphorylation of PI3K and Akt and intensifying the expression of vascular endothelial growth factor (VEGF)-A and VEGF receptor-2 in these cells *in vitro*. In addition, a study using a chick model of the chorioallantoic membrane demonstrated that TFAM clearly promoted the formation of branched blood vessels *in vivo* (Zhu et al., 2018). These results indicate that TFAM may exert proangiogenic activity by activating the VEGF-A/VEGF receptor-2–PI3K/Akt signaling pathway both *in vitro* and *in vivo*.

### Effect on Cerebral Infarction

TFAM (at doses of 30, 60, and 120 mg/kg, i.v.) prolonged the survival time, increased the survival rate, and decreased the MDA content of the cerebral cortex in a mouse model of nitrogen anoxia. In addition, TFAM (at doses of 12, 24, and 48 mg/kg, i.v.) remarkably reduced electroencephalogram activity and the levels of MDA and LDH in rabbits subjected to cerebral ischemia–reperfusion (Guo and Chen, 2002). In addition, TFAM (at doses of 25, 50, and 100 mg/kg, i.g.) markedly decreased the extent of cerebral infarction and increased the contents of LDH and NO in a rat model of occlusion of the right middle cerebral artery (Gao et al., 2002). These findings indicate that TFAM has protective effects against cerebral ischemia–reperfusion injury, and its mechanism of action may be related to the inhibition of free radicals and lipid peroxidation.

Pretreatment with TFAM (at doses of 20, 40, 80, and 160 mg/kg, i.g.) led to a remarkable percentage reduction in the extent of cerebral infarction. Pretreatment with TFAM also significantly reduced the activity of LDH and the MDA content, increased the serum levels of NO, and upregulated the cerebral mRNA expression of inducible nitric oxide synthase (iNOS) in rats with ischemia–reperfusion-induced cerebral infarction subjected to occlusion of the right middle cerebral artery in comparison with nimodipine (2 mg/kg) as a control (Wen and Chen, 2007). These findings indicate that TFAM reduces the extent of cerebral infarction, which is partly due to its antioxidant and anti-inflammatory activities.

### Anti-Crohn’s Disease Activity

TFAM at concentrations of 5, 10, and 15 μg/mL effectively suppressed TGF-β1-induced morphological changes, migration, and invasion; increased the mRNA and protein levels of E­cadherin and ZO-1; decreased the mRNA and protein levels of vimentin and N­cadherin; and reduced the levels of Smad 2/3, p-p38, p-c-Jun N-terminal kinase, and p-ERK_1/2_, which involved the Smad/MAPK signaling pathway. In addition, si-Smad and MAPK inhibitors effectively suppressed the TGF-β1-induced epithelial–mesenchymal transition in IEC-6 cells (Yang B. L. et al., 2018). Thus, a combination of TFAM with si-Smad or MAPK inhibitors had greater inhibitory effects on the TGF-β1-induced epithelial–mesenchymal transition in IEC-6 cells, which suggests that TFAM might represent a novel therapeutic strategy for the treatment of intestinal fibrosis in Crohn’s disease.

### Effect on Bone Loss

The ability of *A. manihot* leaves to inhibit bone loss in ovariectomized rats was investigated by Puel et al. (2005). The results of this study showed that osteopenia was significantly reduced after the administration of the highest dose of the leaves of *A. manihot*, namely 15%, in the diet, although the lowest dose did not produce any effects: bone-sparing effects were produced, which enabled improvements in bone mineral density and bone mineral content (Puel et al., 2005). Therefore, *A. manihot*, as an edible plant, has been used in folk medicine to alleviate symptoms of sex hormone imbalances and can help to reduce bone loss in conditions of estrogen deficiency and thus provide some protection against osteoporosis (Puel et al., 2005). Further study data are required for the development of potential alternative nutraceutical agents.

### Antiplatelet Activity

TFAM at doses of 25, 50, and 100 mg/kg (i.g.) for 3 days notably decreased the weight of thrombi in a rat model of thrombus formation induced by arteriovenous bypass in a dose-dependent manner. Moreover, TFAM at concentrations of 0.025, 0.05, and 0.10 mg/mL displayed dose-dependent inhibitory effects on platelet aggregation induced by collagen in rabbits. In addition, TFAM markedly reduced the resting and CaCl_2_-induced increased concentrations of free intracellular calcium ions in rabbit platelets *in vitro* (Guo et al., 2005). Therefore, TFAM may have wide therapeutic potential for treating various circulatory diseases, such as cerebral infarction.

### Other Activity

The effect of a combination of quercetin and hyperoside (at a dose of 20 mg/kg [i.g.] for 21 days) on the formation of calcium oxalate deposits in rats fed ethylene glycol was studied. The results showed that the quercetin–hyperoside combination remarkably reduced the number of crystal deposits and increased the activities of SOD and CAT in comparison with those in the untreated group (Zhu et al., 2014). Therefore, quercetin in combination with hyperoside may act as a complementary effective drug for preventing stone formation.

### Pan-Assay Interference Compounds

In 2003, high-throughput screening technology as a normal method was established to identify novel lead compounds to increase the number of validated drug targets in drug screening (Baell and Walters, 2014; Baell, 2016; Baell and Nissink, 2018). However, it also introduced many special molecules that interfere with drug screening, such as pan-assay interference compounds (PAINS), which resulted in false-positive assay readouts in pharmacological investigations, and ignored bioactive compounds with real potential (Baell and Walters, 2014), suggesting that the structural context in which PAINS are presented may play an important role for eliciting false-positive activities. Moreover, at least 10 chemical constituent classes have been considered to be PAINS, including rhodanines, phenolic Mannich bases, 1,2,3-aralkylpyrroles, hydroxyphenylhydrazones, alkylidene barbiturates, alkylidene heterocycles, 2-amino-3-carbonylthiophenes, activated benzofurazans, catechols, and quinones. These should be avoided in further studies as they largely reduced the false-positive readouts of the lead compound (Baell and Holloway, 2010). Hence, PAINS have already become a major point for discussion in drug discovery, especially in the fields of medicinal plant research, ethnopharmacology, and natural product research with respect to their pharmacological investigations (Baell and Walters, 2014; Heinrich et al., 2020).

In the present review, flavonoid compounds, as major bioactive ingredients purified from different medicinal parts of *A. manihot*, exhibited a variety of pharmacological activities. However, these activities may have been affected by PAINS; consequently, the results reported should be regarded with skepticism. Simultaneously, the screening approach should be studied carefully during drug screening to reduce the interference of PAINS. In addition, PAINS should be completely eliminated by various methods, including computer prediction and biochemical methods, especially in the early stages of drug research and development. It is worth noting that, in view of the complexity of biological systems and the characteristics of the interactions between drugs and the body, in the process of predicting the effects of different compounds, accurate elimination of PAINS still needs to be specifically investigated in future studies.

## Pharmacokinetics and Metabolism

The metabolites of bioactive flavonoids from *A. manihot* have been investigated over the past decade. Hibifolin, isolated from *A. manihot* flowers, was incubated with human intestinal bacteria, and then four metabolites, namely gossypetin 8-*O*-β-D-4′-deoxy-Δ^4′′^-glucuropyranoside, quercetin, 8-methoxyquercetin, and gossypetin, were detected in the incubation solution on the basis of ultraviolet, MS, and nuclear magnetic resonance data (Xu et al., 2009). Pharmacokinetics studies were carried out after the administration of HKC at a dose of 0.75 g/kg (i.g.) for 10 consecutive days to rats in combination with 1 mg/kg glibenclamide, which was administered before the first time and after the last time that HKC was given. The results of this study showed that the plasma concentration of glibenclamide was markedly reduced when it was coadministered with HKC, and the maximum serum concentration and area under the concentration–time curve (AUC) significantly decreased, which suggests that HKC can decrease the absorption and accelerate the metabolism of glibenclamide by inducing the enzyme cytochrome P450 (Liu Z. X. et al., 2012). Therefore, when glibenclamide and HKC are combined to treat patients with nephropathy, the glibenclamide concentration in plasma should be carefully monitored (Liu Z. X. et al., 2012).

Guo et al. (2010) developed a rapid analytical method using ultra-performance liquid chromatography (UPLC)–quadrupole time-of-flight MS in combination with automated data analysis software to determine the metabolic profiles of flavonoids from *A. manihot* in plasma and urine samples from rats. The results showed that 16 and 38 metabolites were identified in plasma and urine, respectively, in comparison with the corresponding blank samples, which suggests that methylation and glucuronidation after deglycosylation are the main metabolic pathways of flavonoid glycosides (Guo et al., 2010). Xue et al. (2011a) used the same method to determine the metabolic profile of an *A. manihot* extract in intestinal flora. A total of 14 compounds, including six native chemical constituents and eight metabolites, were identified in incubation solutions from humans and rats. Taken together, these findings clearly suggest that flavonoids in *A. manihot* extracts can be metabolized in intestinal bacteria to generate metabolites *via* hydrolysis, hydroxylation, acetylation, methylation, and deoxygenation (Xue et al., 2011a). Xue et al. (2011b) also analyzed potential active agents in the blood and kidney of rats after oral administration of an *A. manihot* extract at a dose of 6.8 g/kg (equivalent to 5.8, 6.0, 12.2, and 5.1 mg/kg isoquercitrin, hyperoside, hibifolin, and quercetin-3′-*O*-glucoside, respectively) using microdialysis in combination with UPLC–quadrupole time-of-flight MS. The results showed that unbound compounds in rat blood included quercetin-3′-*O*-glucoside, hibifolin, hyperoside, myricetin, quercetin, isoquercitrin, and quercetin monoglucuronide, whereas unbound compounds in the kidney included hyperoside, isoquercitrin, and quercetin monoglucuronide (Xue et al., 2011b).

Cao et al. (2010) devised a method that used HPLC to determine the concentrations of quercetin-3′-*O*-glucoside, hyperoside, isoquercitrin, and hibifolin in rat plasma after i.g. and i.p. administration of extracts of *A. manihot* flowers to rats. The results showed that high linearity was achieved in the concentration range of 0.1–8 μg/mL for flavonoids in rat plasma, with a value of *r* > 0.99. The limit of quantification was 0.1 μg/mL and the recovery rate exceeded 70%. The intra- and inter-day relative standard deviations were both less than 15%. Pharmacokinetic parameters of flavonoids differed from each other after i.g. and i.p. administration. The absolute bioavailability of the above flavonoids was 12.9%, 10.8%, 2.2%, 10.2%, and 5.9%, respectively (Cao et al., 2010). Similarly, Guo et al. (2012) compared the effects of the two different administration routes on the brains of rats after i.g. and i.p. administration of hyperoside *in vivo*. Interestingly, pharmacokinetics results showed that hyperoside and its metabolite 3′-*O*-methylhyperoside were detected using UPLC–tandem mass spectrometry (MS/MS) and microdialysis in rat brains after i.p. administration but not after i.g. administration. Moreover, i.p. administration of hyperoside resulted in higher bioavailability and biological activity in rat brains. Furthermore, the forms of hyperoside present in rat brains were hyperoside and its methylated metabolite, with maximum concentrations of 63.78 ng/mL and 24.66 ng/mL, respectively, after i.p. administration at 20 mg/kg (Guo et al., 2012). These results might be used to further study the bioavailability of TFAM and establish a reasonable dosage for clinical use.

In addition, Guo et al. (2016) further investigated the pharmacokinetic profile of the flavonoid fraction of *A. manihot* at a dose of 400 mg/kg after oral administration to normal rats and a rat model of CKD. The results showed that the AUC values for quercetin glucuronide conjugates, isorhamnetin glucuronide conjugates, quercetin sulfate conjugates, and isorhamnetin sulfate conjugates in plasma from normal rats were 459.45, 1153.01, 417.81, and 2475.19 μmol h/L, respectively (Guo et al., 2016). In contrast, the AUC values for quercetin and isorhamnetin were 5.47 and 30.73 μmol h/L, respectively (Guo et al., 2016). Therefore, the AUC values for the glucuronide and sulfate conjugates of quercetin and isorhamnetin were approximately 125 times those for the corresponding aglycones (i.e., quercetin and isorhamnetin), which indicates that glucuronide and sulfate conjugates represent the main circulating forms of the flavonoid fraction of *A. manihot* *in vivo* (Guo et al., 2016). Moreover, the AUC values for isorhamnetin glucuronide conjugates and quercetin sulfate conjugates were 719.65 and 275.49 μmol h/L, respectively, which suggests that few conjugated metabolites were formed in rats with CKD in comparison with normal rats. The AUC_glucuronide/sulfate_/AUC_aglycone_ ratio decreased from 125 to 104, which suggests that the ability to perform phase II metabolism was impaired in rats with CKD (Guo et al., 2016).

## Clinical Settings

DN is one of the most common primary glomerular diseases worldwide and is a major cause of cardiovascular events. It has thus become a major threat to human health and cause of mortality, but effective therapies remain limited and many patients progress to end-stage renal disease (Balakumar et al., 2012). However, the use of traditional Chinese medicines and natural products has attracted the attention of scientists involved in research into kidney disease. The results of the first randomized controlled trial carried out to examine the safety and efficacy of HKC in patients with primary glomerular disease showed that HKC (at a dose of 2.5 g [i.g.[three times per day) significantly decreased 24 h urinary protein after treatment for 24 weeks. This suggests that HKC might be a promising agent for patients with primary kidney disease and moderate proteinuria, especially in CKD stages 1 and 2 (Carney, 2014; Zhang et al., 2014). Li et al. (2017) assessed the efficacy and safety of HKC for treating immunoglobulin A (IgA) nephropathy in a multicenter, prospective, double-blind, and double-dummy randomized controlled clinical trial by enrolling approximately 1600 patients with biopsy-proven IgA nephropathy at 100 centers and following them for up to 48 weeks. The results of this study indicated that HKC (at a dose of 2.5 g [i.g.] three times per day) markedly decreased 24 h urinary protein and the estimated glomerular filtration rate with respect to baseline after treatment for 48 weeks (Li et al., 2017). In addition, two systematic reviews and a meta-analysis were conducted to determine the safety and efficacy of HKC for the treatment of DN and type 2 DN, respectively. The results showed that HKC remarkably reduced 24 h urinary protein, proteinuria, the 24 h urinary protein reduction rate, the urinary albumin excretion rate, and serum creatinine levels; restored a normal urinary albumin excretion rate; and protected kidney function in patients with DN and type 2 DN (Chen et al., 2015; Chen et al., 2016; Shi et al., 2019). The results of these studies demonstrate that *A. manihot* is a potential promising candidate agent for the treatment of patients with CKD (Guo et al., 2015).

## Toxicity Assessment

Although *A. manihot* has been used as a staple food or medicine for a long time, its systematic safety and toxicity remain unclear. Only two tests of the acute toxicity of *A. manihot* have been performed; specifically, by i.g. administration to Swiss albino mice and Wistar rats at a dose of 2.0 g/kg for 14 days. The results found no mortality or significant changes in the eyes; mucous membranes; skin; respiratory, circulatory, autonomic and central nervous systems; or in behavior, food intake, and body weight, which further proved that *A. manihot* is non-toxic (Jain et al., 2009; Pritam et al., 2011). Although these toxicity studies were conducted on animals, no safety assessments have been carried out on dogs, monkeys, or even humans in clinical practice.

## Preparation and Qualitative and Quantitative Analysis

Significantly, flavonoids are considered to be the major effective constituents of *A. manihot*. Lai et al. (2007) devised and validated a method for the simultaneous determination of five flavonols, namely isoquercitrin (1), hibifolin (2), myricetin (3), quercetin-3′-*O*-D-glucoside (4), and quercetin (5), in rat plasma and urine after oral administration of TFAM. Analysis of the plasma and urinary extracts was performed with a YMC-Pack ODS-A C_18_ column and a Thermo ODS-2 Hypersil C_18_ reversed-phase column, respectively. A mobile phase consisting of acetonitrile–0.1% phosphoric acid was employed. HPLC analysis was conducted with different elution gradients. The flow rate was 1.0 mL/min, and the detection wavelength was set at 370 nm. The calibration ranges for flavonols 2–5 in plasma were 0.011–2.220 μg/mL, 0.014–2.856 μg/mL, 0.022–4.320 μg/mL, and 0.028–5.600 μg/mL, respectively. In urine, the calibration ranges for flavonols 1, 2, 4, and 5 were 2.00–16.00 μg/mL, 8.56–102.72 μg/mL, 2.70–21.60 μg/mL, and 3.00–24.00 μg/mL, respectively. The intra- and inter-day relative standard deviations were less than 5.40% and 4.89%, respectively, for plasma and less than 3.96% and 6.85%, respectively, for urine (Lai et al., 2007).

Later, Lai et al. (2009) developed an HPLC method for the simultaneous quantification of seven flavonols, namely quercetin-3-*O*-robinobioside, hyperoside, isoquercitrin, hibifolin, myricetin, quercetin-3′-*O*-glucoside, and quercetin, in *A. manihot* flowers. HPLC separation was performed using a linear gradient at room temperature (25 °C) and a flow rate of 1.0 mL/min with a Thermo ODS-2 Hypersil reversed-phase column connected to a Phenomenex C_18_ guard column. The gradient elution started with a solution of acetonitrile and phosphoric acid (15 : 85, v/v), the acetonitrile content was increased to 40% within 40 min, and the detection wavelength was 370 nm. All seven flavonols were effectively separated and identified. The recovery rate achieved using this method was 94.31–107.08% with a relative standard deviation of ≤3.14%, and high linearity (*R* ^2^ > 0.9996) was achieved for all the analytes (Lai et al., 2009).

In addition, Du et al. (2015a) developed a UPLC–triple-quadrupole MS/MS method to detect 12 nucleotides, nucleosides, and nucleobases present in the roots, stems, leaves, and flowers of *A. manihot* and successfully identified these 12 analytes in different parts of *A. manihot* harvested during 10 growth periods. The results showed that the distribution and concentration of the 12 compounds in the four plant parts occurred in decreasing order as follows: leaves > flowers > stems > roots, which indicates that the leaves and flowers of *A. manihot* might be developed in the future into health products with nutraceutical and bioactive properties. Moreover, this method could be used for the quality control of *A. manihot* leaves and other herbal medicines that are rich in nucleotides, nucleosides, and nucleobases (Du L. Y. et al., 2015). Subsequently, Pan et al. (2017) devised a rapid and highly sensitive UPLC–MS/MS method, which was successfully used for the simultaneous determination of five flavonoids (rutin, hyperoside, isoquercitrin, quercetin, and myricetin) in different parts of *A. manihot* harvested during 10 growth periods. The results indicated that the total contents of these five flavonoids in the roots, stems, leaves, and flowers of *A. manihot* ranged from 2.86 to 123.7 μg/g, 46.39 to 141.0 μg/g, 929.4 to 3096 μg/g, and 10 150 to 19 390 μg/g, respectively, which suggests that the total flavonoids were present in decreasing order as follows: flowers > leaves > stems > roots (Pan et al., 2017).

Wan et al. (2018) developed novel molecularly imprinted polymers (MIPs) based on bifunctional monomers and successfully isolated and identified myricetin from *A. manihot*. The results of this study revealed that these MIPs exhibited high adsorption ability and selectivity toward myricetin. Finally, the MIPs were employed as adsorbents for the solid-phase extraction of myricetin from safflower and the flowers of *A. manihot*. Further analysis was conducted using HPLC–diode array detection. The recovery of myricetin from safflower and the flowers of *A. manihot* ranged from 79.82% to 83.91% and from 81.50% to 84.32%, respectively, which suggests that MIPs can be used for the extraction and separation of myricetin from various complex matrices (Wan et al., 2019).

In a recent study, a highly efficient and ecofriendly extraction method using deep eutectic solvents was devised to isolate bioactive flavonoids from *A. manihot*. The extraction conditions were as follows: solid to solvent ratio, 35 : 1 (mg/mL); extraction time, 30 min; and extraction temperature, 30 °C. Moreover, qualitative and quantitative analyses were carried out using UPLC–MS/MS and HPLC. The results showed that the extraction efficiencies for hyperoside, isoquercitrin, and myricetin under the optimal extraction conditions were calculated to be 11.57 mg/g, 5.64 mg/g, and 1.11 mg/g, respectively, which were much higher than those achieved using traditional extraction solvents (Wan et al., 2019).

## Future Perspectives and Conclusions

As an important traditional medicine in eastern Europe and Asia, the flowers of *A. manihot* have been used in a variety of clinical applications and also as a vegetable by local people for thousands of years. Numerous studies have been conducted and have shown that *A. manihot* flowers exhibit many biological effects, many of which are in accordance with their traditional uses. Flavonoids are used to monitor the quality of *A. manihot*, and the hyperoside content should be a minimum of 0.5% according to the 2015 edition of the *Chinese Pharmacopoeia* (Committee for the Pharmacopoeia of P.R. China, 2015). In traditional Chinese medicine, the flowers of *A. manihot* are mainly applied topically to treat CKD and related inflammatory diseases. In modern medical practice, *A. manihot* flowers exhibited promising renoprotective effects, and thus the medicinal value of *A. manihot* for the treatment of kidney diseases and syndromes has been fundamentally proved. In addition, many studies on chemical constituents and pharmacological effects have validated ethnomedicinal uses of *A. manihot*.

Taken together, *A. manihot* is a valuable medicinal resource with specific biological activities demonstrated in *in vivo* studies. However, there are also some shortcomings and further improvement and research are needed, as follows. (1) As important traditional Chinese medicines, the flowers, roots, stems, leaves, and seeds of *A. manihot* possess a wide range of biological properties. However, only the flowers of *A. manihot* are regarded as medicinal parts in the commonest modes of application, whereas the roots, stems, leaves, and seeds are discarded and burned, which undoubtedly causes serious environmental pollution and resource waste and also results in sharp reductions in resources of *A. manihot*. Further phytochemical studies of these parts are necessary, especially with regard to their polysaccharide constituents, which are used as stabilizers and thickeners in the food industry. (2) The flavonoids, as major active components, have received attention from scientists because of their renoprotective activity and high nutritional value, and further investigations into these active constituents should be a priority. Moreover, the mechanisms of action responsible for the anti-DN, anticonvulsant, antioxidant, antidepressant, anti-inflammatory, cardioprotective, and neuroprotective effects of these active flavonoids need to be scientifically investigated in depth to clarify the precise mechanisms responsible for their various activities both *in vivo* and *in vitro*. Meanwhile, efforts should be put into the elimination of false negatives, such as PAINS. (3) Although the flowers of *A. manihot* are listed in the 2015 edition of the *Chinese Pharmacopoeia* and are widely used in clinical practice, animal experiments and randomized clinical trials involving this plant and its derivatives are still in the exploratory stage, and the precise mechanisms responsible for their therapeutic effects remain unknown. Therefore, multicenter clinical trials with large samples need to be conducted and high-level clinical evidence needs to be obtained urgently to explain the mechanisms of action and to identify potential lead compounds for further development of *A. manihot* constituents and guarantee their safety and effectiveness in clinical practice. (4) HKC has been demonstrated to effectively alleviate proteinuria and improve kidney function in CKD in clinical practice and flavonoids are responsible for protecting renal function. However, the dosages of HKC used (7.5 g/day for an adult by oral administration) are still relatively high, which might affect patient compliance. Therefore, more advanced purification techniques need to be rapidly developed and more in-depth studies should be conducted to purify and enrich the flavonoids and thus enable a decrease in the dosage of the respective remedy obtained from *A. manihot*. For example, Box–Behnken response surface methodology experiments and static adsorption–desorption experiments should be employed to identify the optimal process parameters for the extraction of flavonoids, which would enable the further purification and enrichment of flavonoids, improve patient compliance, and eventually lead to effective treatment in clinical practice. (5) Importantly, large numbers of amino acids and nucleotides have been demonstrated to be present in the stems and leaves of *A. manihot*. Hence, the stems and leaves of *A. manihot* might be developed in the future into health products that possess nutraceutical and biological properties. (6) In regard to its toxicity, some studies have suggested that the *A. manihot* has low toxicity. To conclude, future investigations should include the identification of any side effects or toxicity. Meanwhile, more systematic and comprehensive investigations of pharmacokinetics, metabolism, and clinical settings will be indispensable to demonstrate the safety and efficacy of the main biologically active compounds from different medicinal parts of *A. manihot* before proceeding to the development of pharmaceutical formulations. (7) Future studies on *A. manihot* should focus on establishing the links between traditional uses, bioactive ingredients, and claimed pharmacological properties. We expect to obtain molecules with new skeletons and new activities from different parts of *A. manihot*.

## Author Contributions

QW, DL, and ZG obtained the literatures. FL, HL, and NZ wrote the manuscript. FL, HL, and YY revised the manuscript and NZ contributed ideas and edited the manuscript. All authors contributed to the article and approved the submitted version.

## Funding

This study was financially supported by the National Natural Science Foundation of China (no. 81473399).

## Conflict of Interest

The authors declare that the research was conducted in the absence of any commercial or financial relationships that could be construed as a potential conflict of interest.

## Abbreviations

CKD, chronic kidney disease; DN, diabetic nephropathy; HKC, Huangkui capsule; TCM, traditional Chinese medicine; HPLC, high-performance liquid chromatography; MS, mass spectrometry; TNF-α, tumor necrosis factor-α; TGF-β1, transforming growth factor-β1; p38MAPK, p38 mitogen-activated protein kinase; BUN, blood urea nitrogen; STZ, streptozotocin; Akt, serine–threonine kinase; PPAR-α/-γ, peroxisome proliferator-activated receptors-α; Alb, albumin; IL-1/1β/2/6/17/22, interleukin-1/1β/2/6/17/22; UP, urine protein; Scr, serum creatinine; NOX-1/2/4, NADPH oxidase 1/2/4; α-SMA, α-smooth muscle actin; p-ERK_1/2_, phosphorylation–extracellular signal-regulated kinase; CRF, chronic renal failure; PCNA, proliferating cell nuclear antigen; GBM, glomerular basement membrane; mTOR, mammalian target of rapamycin; p70S6K, 70-kDa ribosomal protein S6 kinase; PI3K, phosphatidylinositol-3-kinase; TFAM, total flavones of *A. manihot*; AGEs, advanced glycation end-products; TACE, tumor necrosis factor-a converting enzyme; ROS, reactive oxygen species; NLRP3, nucleotide-binding oligomerization domain, leucine-rich repeat, and pyrin domain containing-3; IR, ischemia–reperfusion; AKI, acute kidney injury; CAT, catalase; SOD, superoxide dismutase; T-AOC, total antioxidant capacity; MDA, malondialdehyde; GPx, glutathione peroxidase; DSS, dextran sulfate sodium; PSD, poststroke depression; HBV; hepatitis B virus; ALT, alanine aminotransferase; AST, aspartate aminotransferase; ALP, alkaline phosphatase; γ-GT, gamma glutamyltransferase; GST, glutathione transferase; TBIL, total bilirubin; DBIL, direct bilirubin; TBA, total bile acid; NO, nitric oxide; LDH, lactate dehydrogenase; CK, creatinine kinase; CAM, chick chorioallantoic membrane; VEGF, vascular endothelial growth factor; MCA, middle cerebral artery; EMT, epithelial–mesenchymal transition; BMD, bone mineral density; BMC, bone mineral content; OPA1, optic atrophy 1; NMR, nuclear magnetic resonance; UPLC-Q-TOF/MS, ultra-performance liquid chromatography/quadrupole time-of-flight mass spectrometry; UPLC-MS/MS, ultra-high-performance liquid chromatography coupled with tandem mass spectrometry; ESRD, end-stage renal disease; eGFR, estimated glomerular filtration rate; HPLC-DAD, high-performance liquid chromatography-diode array detection; MIPs, molecularly imprinted polymers; TST, tail suspension test; FST, forced swimming test; BDNF, brain-derived neurotrophic factor; TrkB, tyrosine receptor kinase; p-eEF2, phosphorylation eukaryotic elongation factor 2; CREB, cAMP-response element-binding protein.
